# Genotype performance, biochemical and anatomical response of two mango rootstocks to glycine betaine and algae extract under salinity stress

**DOI:** 10.1186/s12870-026-09893-w

**Published:** 2026-09-25

**Authors:** Mohamed S. Gawish, Atef M. Abo-Ogiala, Elsayied A.E. Abdel-Aziz, Essam-Eldin A. Ahmed, Mohamed S. Abo El-Enien, Abdel-Moety Salama

**Affiliations:** 1 Horticulture Department, Faculty of Agriculture, Damietta University, New Damietta, 34517 Egypt; 2 The Center for Excellence in Research of Advanced Agricultural Sciences (CERAAS), Faculty of Agriculture, Damietta University, New Damietta, 34517 Egypt; 3 Horticulture Department, Faculty of Agriculture, Tanta University, 31527 Tanta, Egypt; 4 Agriculture Botany Department, Faculty of Agriculture (Cairo), Al-Azhar University, Nasr City , Cairo, 11765 Egypt; 5 Horticulture Research Institute (HRI), Agricultural Research Center, Giza, 12619, Egypt; 6 Horticulture Department, Faculty of Agriculture, Kafrelshaikh University, Kafr El-Sheikh, 33516, Egypt

**Keywords:** Mango rootstocks, Salinity stress, Oxidative stress, Antioxidant enzymes, Glycine betaine, Seaweed algae extracts, Sokkary, Gahrawy, Climate resilience

## Abstract

**Background:**

Salinity is a major abiotic constraint limiting mango (Mangifera indica L.) production, particularly in arid and semi-arid regions. This study evaluated the effects of foliar application of glycine betaine (GB) and algae extract (AE) on the growth, physiological, biochemical, and anatomical responses of two mango rootstocks, Sokkary and Gahrawy, irrigated with tap water, 1500 ppm, or 3000 ppm seawater.

**Results:**

Salinity significantly reduced plant height, stem diameter, leaf area, chlorophyll content, and the K⁺/Na⁺ ratio, while increasing malondialdehyde (MDA), hydrogen peroxide (H₂O₂), and antioxidant enzyme activities. At 3000 ppm, untreated plants reached only 24–25 cm in height, whereas GB application in Gahrawy and AE application in Sokkary substantially alleviated this inhibition, restoring height to 42.25 and 40.00 cm, respectively. Gahrawy exhibited greater intrinsic salinity tolerance, with higher K⁺ retention (2.174%), endogenous glycine betaine (2.91 µmol g⁻¹ DW), and antioxidant capacity (40.23% DPPH scavenging). In contrast, Sokkary responded more strongly to biostimulant application, particularly GB under severe salinity. GB improved osmotic adjustment, ion homeostasis, and chlorophyll stability, increasing plant height to 50.0 cm in Gahrawy at 1500 ppm and reducing leaf Na⁺ accumulation by up to 51% at 3000 ppm. AE enhanced antioxidant activity (53.0% DPPH scavenging), leaf expansion, and vascular development. Anatomical analyses revealed genotype-specific structural adaptations associated with salinity tolerance.

**Conclusions:**

Salinity tolerance depended on the interaction among genotype, salinity level, and biostimulant treatment. GB and AE effectively alleviated salt-induced damage through complementary physiological and anatomical mechanisms, supporting their use as sustainable strategies to improve mango production under saline conditions.

## Introduction

Mango (Mangifera indica L.) is a globally significant fruit crop, ranking as the second most cultivated tropical fruit and the sixth major fruit crop worldwide [1]. In 2024, global mango production is estimated to exceed 22 million MT, with Egypt contributing substantially to its cultivated area, which reaches approximately 78,848 hectares, and annual production is about 850,115 tons, making mango the third most important fruit crop in the country. However, mango productivity is increasingly threatened by abiotic stresses, particularly soil salinity, which is a growing concern in arid and semi-arid regions such as Egypt [1, 2]. Salinity is a major abiotic stressor threatening mango productivity, especially in arid and semi-arid regions like Egypt, where improper irrigation practices and climate change exacerbate the problem [3–6]. Salinity stress impairs mango growth and yield by reducing plant height, leaf area, chlorophyll content, and nutrient uptake, while increasing leaf abscission and oxidative damage [5, 7]. Mango is generally considered salt-sensitive, particularly at the seedling stage, with symptoms including leaf scorching, curling, and, in severe cases, plant death [8, 9]. Physiologically, salinity induces ionic toxicity, osmotic stress, and oxidative stress, leading to reduced productivity and fruit quality [10, 11]. Rootstock selection is a critical strategy for mitigating salinity effects in mango cultivation. Significant variation exists among mango rootstocks in their ability to tolerate salinity. For example, polyembryonic genotypes such as’Turpentine’, ‘Olour’, ‘Nekkare’, and ‘Kurukkan’ have demonstrated higher tolerance by restricting toxic ion uptake, accumulating osmoprotectants like proline and glycine betaine, and enhancing antioxidant responses [2, 10, 12] Studies show that salt tolerant rootstocks like ‘13/1’ delay leaf abscission and maintain better growth under salt stress compared to more sensitive types like ‘Gomera-3‘ [3, 12]. The use of salt-tolerant rootstocks is increasingly recognized as an effective and sustainable approach to maintaining mango productivity in saline soils [1, 3, 4, 10, 12]. In addition to rootstock selection, natural biostimulants such as seaweed algae extracts and glycine betaine have emerged as promising tools for alleviating salinity stress. Seaweed extracts, rich in bioactive compounds, enhance plant growth, photosynthetic efficiency, and antioxidant defenses under salinity, as demonstrated in mango and other crops [13, 14]. Foliar application of seaweed extracts has been shown to significantly improve vegetative growth, yield, and fruit quality in mango, especially under stress conditions.

Glycine betaine, a naturally occurring osmoprotectant, stabilizes cellular structures and enzymes, improving growth and yield in salt-affected plants, including mango [4, 7, 8]. Combined applications of glycine betaine and seaweed extracts have been particularly effective in mitigating the negative impacts of salt stress on mango seedlings, improving growth parameters, chlorophyll content, and nutrient balance [4, 5, 15]. Sustaining mango productivity in Egypt and worldwide under increasing salinity stress requires integrated approaches. These include the use of salt-tolerant rootstocks, application of natural biostimulants such as seaweed extracts and glycine betaine, and improved irrigation and soil management practices [2, 3, 13, 15, 16]. Such strategies not only enhance plant resilience and yield but also contribute to more sustainable and environmentally friendly mango cultivation systems. The synergistic application of seaweed extracts and glycine betaine represents an innovative integrated approach to enhancing mango resilience under salinity stress, potentially offering complementary mechanisms of action that address multiple facets of salt-induced damage [4, 7, 13–15, 17]. However, a comprehensive understanding of their interactive effects on mango physiological responses and biochemical adaptation parameters under field conditions remains limited. To our knowledge, this is the first factorial study to comprehensively evaluate the individual and combined effects of glycine betaine (GB) and seaweed algae extract (AE) on salinity tolerance in two genetically distinct mango rootstocks, ‘Sokkary’ and ‘Gahrawy’. We hypothesized that GB and AE would alleviate salinity-induced damage through complementary and genotype-dependent mechanisms, while the rootstock genotype would modulate the magnitude of their beneficial effects. Accordingly, this study aimed to assess the potential of GB and AE, applied individually and in combination, to mitigate salinity-induced constraints, with particular emphasis on growth performance, physiological function, ionic balance, biochemical defense responses, and anatomical adaptations.

### Materials and methods

The present investigation was carried out during two successive seasons 2024/2025 at the nursery of agriculture faculty, Damietta university, Egypt.

#### The plant material

A field study was carried out at the Faculty of Agriculture nursery, Damietta University, Egypt, over two consecutive growing seasons (2024–2025) on two mango rootstocks under salinity stress for 5 months each season. These rootstocks namely “Gahrawy” is planted and expanded in Damietta governorate especially at Om EL-Reda village and the second one is Sukkary. Mango fruits of Sukkary and Gahrawy were brought from a private orchard in Om EL- Reda village at Damietta governorate. Seeds were freshly extracted from ripe fruits and washed twice in tap water and sown at the end of August and September for ‘Sukkary’ and ‘Gahrawy’ cultivars, respectively. The difference in sowing date between the two cultivars (August for ‘Sukkary’ and September for ‘Gahrawy’) reflects the inherent difference in fruit maturity timing, and consequently in the overall developmental schedule, between two cultivars. The experiment was designed so that treatments (salinity stress application) commenced once both cultivars reached the same phenological growth stage, rather than the same calendar date, thereby ensuring a fair comparison between the cultivars under physiologically equivalent conditions. The collected seeds were sown in plastic pots (20 cm) filled with peat moss and sand at a ratio of 1:2v/v, respectively (one seed for each). The bags were watered regularly. Two months after sowing, the nucellar seedlings of uniform growth vigor were selected and transplanted singly into perforated 30 cm pots filled with prescribed medium. The transplanted seedlings were acclimatized for sixty days. Seedlings were fertilized bi-weekly with 100 ml of a solution containing NPK (0.5 g L⁻¹) + humic acid (3 g L⁻¹) throughout the acclimatization and experimental periods. The same fertilization regime was applied uniformly to all treatments, ensuring that any observed effects of GB and AE are attributable to the biostimulants rather than differential nutritio. After the transplantation with four months, the healthy seedlings were classified into nine similar groups (12 seedlings for each).

### Treatment application

Each group was arranged into three replicates (4 seedlings for each) and then subjected to one of the following treatments during both two seasons: Tap water irrigation as control (T1); Tap water irrigation + spraying Glycine betaine at 50 mmol L^− 1^(T2); Tap water irrigation + spraying algae extracts at 1 g L^− 1^(T3); Diluted sea water irrigation at 1500ppm(T4); Diluted sea water irrigation at 1500ppm + spraying Glycine betaine at 50 mmol/L(T5); Diluted sea water irrigation at 1500ppm + spraying algae extracts at 1 g L^− 1^(T6); Diluted sea water irrigation at 3000ppm (T7); Diluted sea water irrigation at 3000ppm + spraying Glycine betaine at 50 mmol/L (T8) and Diluted sea water irrigation at 3000ppm spraying algae extracts at 1 g/L^− 1^(T9). Each treatment combination consisted of three replicates, with four seedlings per replicate (total of 12 seedlings per treatment), and 108 seedlings in total per season (9 treatments × 12 seedlings) for each expremintal season.

Foliar treatments using two anti-salinity compounds were applied with a small pressure pump, with Tween 20 (0.5%) added as a surfactant. The spray solution was applied to the point of complete leaf wetting (runoff). These sprays were carried out monthly throughout the experiment. Irrigation was provided weekly (500 ml per pot) across all growth cycles from autumn through summer. Salinity levels were prepared by diluting Mediterranean Sea water with tap water, applied at regular weekly intervals from the start to the end of the study. To prevent osmotic potential buildup from salt accumulation, each pot was flushed with 500 mL of tap water every three weeks [18]. It should be noted that the reported values (ppm) represent the nominal concentration of the applied irrigation solution rather than a fixed electrical conductivity (EC) of the growing medium. The irrigation regime was designed to include cycles of saline irrigation followed by periodic freshwater flushing every three weeks, in order to simulate a pulsed/intermittent salinity exposure pattern analogous to the fluctuations in soil salinity caused by intermittent rainfall and natural leaching events under field conditions, rather than imposing a constant and unrealistic salinity level on the plants throughout the experimental period. Accordingly, the plant responses recorded in this study reflect adaptation to a dynamic and intermittent salinity exposure pattern, which enhances the ecological realism and field applicability of the findings. Glycine Betaine was sourced from Loba Cheme, India, while the algae extract was used as a commercial product called Alga Stone, purchased from Milestone Fertilizer Company, Egypt, containing alginic acid 20%, amino acids 4%, mannitol 4%, potassium 20%, and organic matter derived from seaweed extracts 20%.

### Seedling rootstocks morphological under salinity stress

#### Seedling height and stem diameter

At the end of the study, rootstock height was recorded in centimeters, shoot number was determined, and stem diameter was measured in millimeters using a digital caliper (Model 500, Fuzhou, China).

#### Rootstock leaves number, rootstock leaf thickness, and rootstock leaf area )

The total number of fully developed leaves per plant per replication was counted for each rootstock. Leaf thickness (LT) in millimeters was measured using a digital caliper (Model 500, Fuzhou, China). Maximum leaf length and width were recorded, and leaf area (LA) in cm² was computed using the formula established by [19]: Leaf area (cm²) = 0.60 × (LL × LW) − 1.05, where LL is leaf length and LW is leaf width.

#### Leaf optical quality

Visual leaf damage was scored at the end of the experiment on a scale of zero to five, following the ranking index of Sun et al.[20]. A score of zero indicated plant death; one, severe visible damage (> 90%); two, moderate visible damage (50–90%); three, mild visible damage (20–50%); four, minimal visible damage (< 20%); and five, no visible damage.

### Chemical analysis of mango rootstocks

#### Analysis of potassium(K) and sodium (Na) levels

Potassium (K) and sodium (Na) in plant tissue were determined following the dry ash procedure of Chapman and Pratt [21](Chapman and Pratt 1961) as described by Estefan [22](Estefan et al. 2013). A 0.5–1.0 g of dry ground plant sample was weighed into a porcelain crucible, placed in a muffle furnace, and the temperature was gradually raised to 550 °C and maintained for 5 h. After cooling, the ash was dissolved in 5 mL of 2 N HCl, brought to 50 mL with deionized water, mixed thoroughly, and allowed to stand for 30 min. The solution was then filtered through Whatman No. 42 filter paper, discarding the first portions of the filtrate. A series of standard solutions was prepared and calibration curves were constructed for both elements. K and Na concentrations in the extracts were measured by flame photometry at emission wavelengths of 767 nm and 589 nm, respectively. Final concentrations were calculated from the calibration curves and expressed relative to the total extract volume and dry plant weight (Estefan, 2013).

### Total chlorophyll

Chlorophyll content (SPAD): Measured non-destructively using SPAD chlorophyll meter (SPAD-502, Konica Minolta) on mature leaves, with readings taken from multiple positions and averaged.

#### Estimating the proline content

Proline was quantified by homogenizing 0.5 g of fresh mango rootstock leaves in 5 mL of 3% sulfosalicylic acid (w/v), followed by centrifugation at 10,000 rpm for ten minutes. An aliquot of 2.0 mL of the resulting supernatant was combined with toluene and ninhydrin reagent to determine proline concentration [23]. Absorbance was then read at 515 nm using a UV-1800 UV–Vis spectrophotometer (Shimadzu, Japan), and proline content was expressed as mg g⁻¹ fresh weight using an L-proline standard curve.

#### Antioxidant capacity (DPPH%)

Antioxidant activity was assessed using a slightly adapted version of the Blots[24] approach. With mango leaf extracts prepared in ethanol and ascorbic acid serving as the reference antioxidant. Equal volumes of plant extract and DPPH solution (60 µM) were combined in a test tube and incubated at room temperature in the dark for 30 min. Absorbance was then recorded at 517 nm using a UV-1800 UV–Vis spectrophotometer (Shimadzu, Japan). Radical scavenging activity was calculated as: DPPH scavenging activity % = (A0 − A1) / A0 × 100, where A0 is the absorbance of the control and A1 is the absorbance of the sample. IC50 was derived from a plot of inhibition percentage against concentration.

#### Non-enzymatic antioxidant compounds (phenols & flavonoids)

Antioxidant compounds, total phenols, and flavonoids were extracted using methanol according to Kosem et al.[25]. Total phenol content was measured by mixing 0.050 mL of alcoholic leaf extract with 0.40 mL of Folin–Ciocalteu reagent and allowing the mixture to stand for three minutes. After adding 0.80 mL of 10% Na₂CO₃, the reaction was incubated in darkness for two hours at room temperature, and optical density was read at 765 nm using a UV-1800 UV–Vis spectrophotometer (Shimadzu, Japan). Results were expressed as mg gallic acid equivalent (GAE) g⁻¹ dry weight using a gallic acid standard curve.

Total flavonoids were determined following Marinova et al.[26]. One milliliter of the alcoholic leaf extract aliquot was mixed with 4 mL of distilled water and 0.3 mL of 5% NaNO₂. After five minutes at 25 °C, 0.3 mL of 10% AlCl₃ was added, and the mixture was further incubated for six minutes at 25 °C before adding 2.4 mL of distilled water and 2 mL of 1 eq/L sodium hydroxide. Absorbance was measured at 510 nm, and total flavonoid content was expressed as mg quercetin equivalent g⁻¹ dry weight.

#### Estimation of glycine betaine (GB)

The glycine betaine content was determined using the method described by Grieve and Grattan [27]. Initially, 0.25 g of dry leaf tissue was placed in a test tube with 10 mL of distilled water and shaken for 24 h. After filtering the solution through Whatman filter paper, 0.25 mL of the filtrate was combined with 0.25 mL of 2 N sulfuric acid in a test tube and placed in an ice bath for one hour. Next, 0.2 mL of potassium iodide (KI-I2) reagent was added, and the mixture was stored in the refrigerator at 4 °C for 16 h. Following this, the samples were centrifuged at 6000 rpm for 25 min, resulting in two phases. The upper phase was discarded, and the lower phase was treated with 6 mL of dichloroethane and vortexed several times. Finally, the absorbance of the resulting samples was measured at 365 nm using a spectrophotometer. The glycine betaine content in the leaf samples was calculated and reported as milligrams per gram of dry weight.

#### Hydrogen peroxide (H_2_O_2_ ) and malondialdehyde (MDA) accumulation

For H₂O₂ determination, 1 g of fresh leaves was mixed with 5 mL of acetone and centrifuged at 6,000 rpm at 4 °C for 15 min, following the procedure of [28]. One milliliter of the extract was combined with 0.2 mL of NH₃ and 0.1 mL of TiOSO₄ (5%), then centrifuged at 6,000 rpm at 4 °C for ten minutes. The resulting pellet was dissolved in 3 mL of 10% H₂SO₄ (v/v) and re-centrifuged at 5,000 rpm at 4 °C for ten minutes. The absorbance of the supernatant was measured at 410 nm, and the H₂O₂ concentration was expressed as mmol min⁻¹ g⁻¹ fresh weight.

MDA content was determined from approximately 2.5 g of mango rootstock leaves. The ground sample was combined with 500 µL of butylated hydroxytoluene (BHT) (2%, w/v), metaphosphoric acid (HPO₃), and thiobarbituric acid (TBA) in ethanol (5%, w/v). The influence of thiobarbituric acid reactive substances ranging from 0 to 2 mM was assessed against 1,1,3,3-tetraethoxypropane (0–1 mM), which served as an MDA equivalent. Absorbance was recorded at 532 nm using a UV–Vis spectrophotometer [29].

#### Assessment of enzyme activity

Peroxidase (POD), ascorbate peroxidase (APX), and polyphenol oxidase (PPO) were extracted by immersing known amounts of frozen mango leaves in ice-cold phosphate buffer (pH 7, 0.02 M) and centrifuging at 12,000 rpm for 20 min at 4 °C [30]. After a 10-minute heating step, enzymatic activity was halted with 2 mL of an acid reagent (5% dichromate/acetic acid, 1:3 v/v), and optical density was read at 610 nm. APX (APX, EC 1.11.1.11) activity was assessed by monitoring absorbance decline at 290 nm using a UV-1800 UV–Vis spectrophotometer (Shimadzu, Japan), with an extinction coefficient of 2.8 mM cm⁻¹ [31]. The reaction was initiated by adding 0.050 mL enzyme extract, 0.5 mL phosphate buffer, 0.075 mL of 2 mM H₂O₂, and 100 µL of 0.5 mM ascorbate, with APX activity reported as ascorbate mmol min⁻¹ g⁻¹ fresh weight.

POD (EC 1.11.1.7) activity was measured using guaiacol as the electron donor and H₂O₂ as the substrate. The reaction mixture consisted of 1% (v/v) guaiacol, 0.3% H₂O₂, and 50 mM sodium phosphate buffer at pH 6.4. Following addition of 200 µL of enzyme extract to 2.8 mL of the reaction mixture, absorbance at 460 nm was recorded every 30 s over three minutes. One unit of POD activity was defined as an absorbance increase of 0.01 min⁻¹ per mg of protein at 460 nm.

Polyphenol oxidase (PPO, EC 1.10.3.1) activity was determined according to the method described by Oktay et al. [32] with slight modifications. The enzymatic extract was prepared by grinding 0.5 g of frozen fresh leaf tissue in ice-cold sodium phosphate buffer (0.1 M, pH 6.8) containing 1% (w/v) polyvinylpyrrolidone (PVP), followed by centrifugation at 12,000 rpm for 20 min at 4 °C. The resulting supernatant was used as the crude enzyme source. The reaction mixture consisted of 2.9 mL of 50 mM sodium phosphate buffer (pH 6.8) containing 10 mM catechol as substrate, to which 0.1 mL of enzyme extract was added. The increase in absorbance was monitored at 420 nm every 30 s for 3 min using a UV-1800 UV–Vis spectrophotometer (Shimadzu, Japan). One unit of PPO activity was defined as an increase in absorbance of 0.001 min⁻¹ per mg of protein, and results were expressed as Unit mg⁻¹ protein min⁻¹.

#### Anatomical investigation

Leaf anatomical analysis was conducted 150 days after treatment initiation during the second growing season, with small leaf samples (5 mm × 5 mm) collected from three representive seedlings of the treatments. Each sample was excised from the midrib at the midpoint of the fourth leaf from the shoot apex. Samples were fixed in FAA fixative for 48 h, washed with sterile water, and dehydrated through a graded ethanol series, followed by clearing through progressively increasing xylene concentrations (3:1, 1:1, 1:3, and pure xylene), before embedding in paraffin wax with a melting point of 52–54 °C. Sections of 10–15 μm thickness were cut with a rotary microtome, double-stained with safranin and light green, cleared in clove oil, and mounted in Canada balsam following the protocol of [33]. ). Microscopic examination was performed using an Olympus CX41 light microscope (Davao City, Philippines) fitted with a TUCSEN digital camera (USB2, H Series, Fuzhou, China). Five sections from three seedlings per treatment (*n* = 15 sections per treatment).were evaluated for cuticle thickness, total leaf blade thickness, midrib zone thickness, palisade and spongy mesophyll dimensions, and the length and width of the main vascular bundle, all expressed in micrometers.

#### Statistical analysis

The current study included a statistical examination of the (mean) data for the two successful seasons. The Co-Stat software package, Version 6.303 (789 Lighthouse Ave PMB 320, Monterey, CA, 93940, USA), was utilized to analyze the data using a complete randomized block design (CRBD) three-ways analysis with three factors (Rootstock genotypes x Salinity levels x Biostimulants Spraying) and three replications. Each treatment combination consisted of three replicates, with four seedlings per replicate (total of 12 seedlings per treatment), and 108 seedlings in total per season (9 treatments × 12 seedlings) for each expremintal season. Duncan’s multiple range test was used to compare the means of all analyzed treatment results, with a significance level of *p* ≤ 0.05. Pearson correlation coefficients (*r*) as descriptive by [34]. All data were tested for bivariate normality prior to analysis, and significance was assessed at *p* < 0.05. PCA was performed on the full standardized dataset to ensure equal weighting of traits with disparate measurement scales. Eigenvalue decomposition of the resulting correlation matrix yielded orthogonal principal components (PCs), each capturing a decreasing proportion of total data variance. Two complementary criteria descriptives by [35–37] were applied. Component loadings the correlations between each original trait and each PC were used to assign biological meaning to the retained components, and biplot visualization was employed to display sample scores and trait loading vectors simultaneously.

## Results

### Growth and Morphological Parameters

**Fig. 1:**
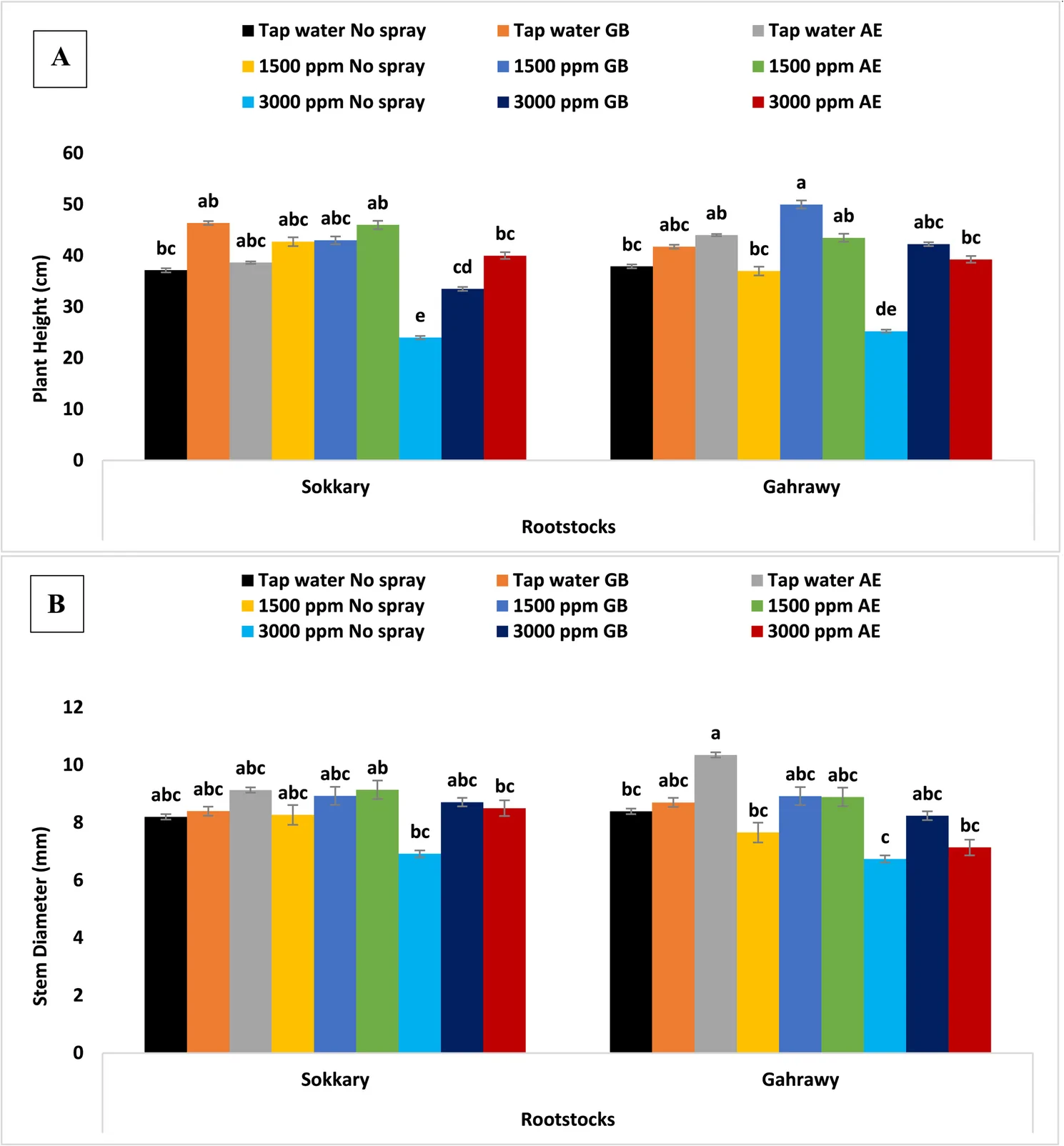
Interaction Effect of salinity levels and spraying treatments of glycine betaine (GB) and seaweed algae extracts AE on plant height (cm) (**A**) and stem diameter (mm) (**B**) of mango rootstocks seedlings

Data presented in Table 1 as main effects and Fig. 1-A as interaction effects showed that rootstock alone had no significant effect on plant height, with comparable mean values for Sokkary (39.04 cm) and Gahrawy (40.09 cm). In contrast, salinity significantly affected plant height, with the highest value recorded at 1500 ppm (43.70 cm), followed by tap water (40.95 cm), while severe salinity at 3000 ppm reduced height to 34.04 cm. Both anti-saline treatments alleviated this growth inhibition, increasing plant height to 42.81 cm with GB and 41.89 cm with AE, compared with 34.00 cm in untreated plants. The interaction effects revealed clear genotype-specific responses. Under moderate salinity, Gahrawy treated with GB reached the maximum height (50.00 cm), representing a substantial improvement over the untreated plants. Under severe salinity, GB with Gahrawy (42.25 cm) and AE with Sokkary (40.00 cm) markedly alleviated growth suppression, indicating differential compatibility between rootstock genotype and biostimulant.

**Table 1:** Main effects of the investigated factors on vegetative and nutritional status of mango rootstocks under salinity conditions

Treatments	Plant height (cm)	Stem Diameter (mm)	Leaf Number	Leaf area (cm)^2^	Leaf thickness (mm)	Leaf optical quality index	Total green color (SPAAD)	K%	Na%
Rootstocks									
Sokkary	39.04	8.49	17.18b	71.20	0.286a	4.09b	57.84b	1.488b	1.335a
Gahrawy	40.09	8.37	18.52a	70.71	0.224b	4.33a	60.74a	2.174	1.266b
F. ≤ 0.05	**ns**	**ns**	*	**ns**	*	*	*	*	*
Salinity Levels									
Tap Water	40.95a	8.86a	18.50a	75.16a	0.260a	4.88a	63.35a	1.521c	0.985c
1500 ppm	43.70a	8.68a	18.50a	72.75a	0.270a	4.41b	59.80b	1.905b	1.262b
3000 ppm	34.04b	7.75b	16.48b	64.95b	0.240b	3.33c	54.62c	2.066	1.656a
F. ≤ 0.05	*	*	*	*	*	*	*	*	*
Anti-Saline									
Non	34.00b	7.70b	16.97b	65.70b	0.244b	3.88b	57.12b	1.720c	1.463a
Glycine Betaine	42.81a	8.65a	18.30a	69.81b	0.261a	4.33a	60.80a	1.987a	1.234b
Algae Extracts	41.89a	8.95a	18.28a	77.35a	0.260a	4.41a	60.04a	1.786b	1.206b
F ≤ 0.05	*	*	*	*	*	*	*	*	*

Note: * and ** indicate significant and highly significant differences, respectively

For stem diameter Table 1; Fig. 1-B, rootstock alone did not significantly affect this trait, with mean values of 8.49 mm for Sokkary and 8.37 mm for Gahrawy. Salinity significantly reduced stem diameter, particularly at 3000 ppm (7.75 mm). Both biostimulants improved stem development, with AE producing the highest overall mean (8.95 mm), followed by GB (8.65 mm), compared with 7.70 mm in untreated plants. Interaction effects further demonstrated genotype-dependent responses. Under tap water, AE produced the greatest stem diameter in Gahrawy (10.35 mm), whereas at 3000 ppm, GB maintained a higher diameter in Sokkary (8.71 mm). Conversely, AE resulted in a marked reduction in Gahrawy (6.14 mm), emphasizing that biostimulant effectiveness depended on both rootstock genotype and salinity intensity.

**Fig. 2:**
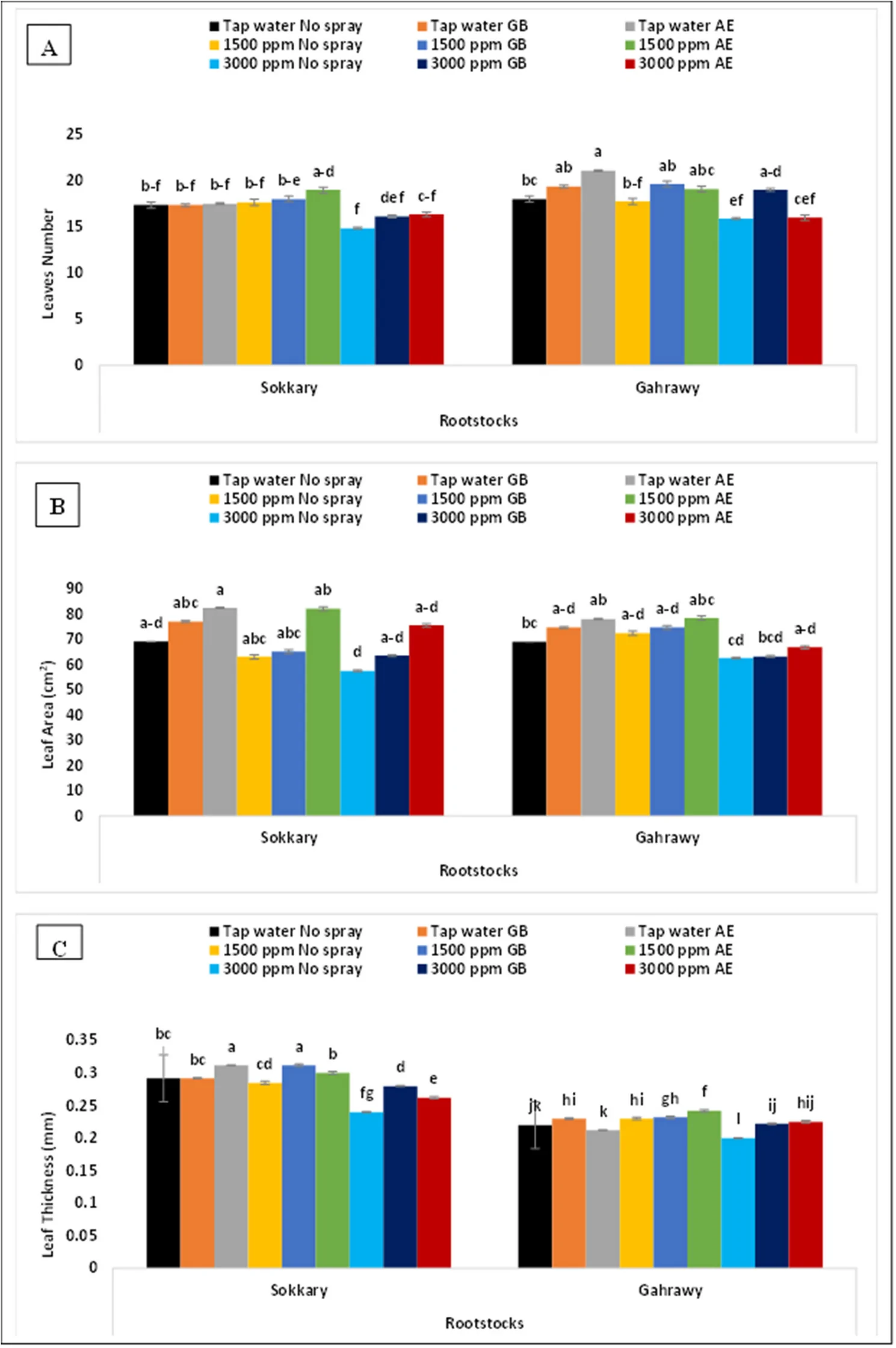
Interaction Effect of salinity levels and spraying treatments of glycine betaine (GB) and seaweed algae extracts AE on Leaves number (**A**), Leaf area (cm)^2^ (**B**) and leaf thickness (mm) (**C**) of mango rootstock seedlings

Data illustrated in Table 1; Figs. 2 A, B and C show that, leaf traits exhibited strong rootstock and treatment interactions. Gahrawy produced significantly more leaves (18.52) than Sokkary (17.18), whereas Sokkary had thicker leaves (0.286 mm compared to 0.224 mm). Salinity significantly reduced leaf number at 3000 ppm (16.48 leaves) and progressively decreased leaf area (from 75.16 cm² under tap water to 64.95 cm² at 3000 ppm). Both GB and AE increased leaf number (~ 18.3 leaves), while only AE significantly enhanced leaf area (77.35 cm²). As for their interactive patterns showed coordinated morphological adjustments. Under tap water, AE application maximized leaf production in Gahrawy (21.15 leaves) and leaf area in Sokkary (82.57 cm²). At 1500 ppm, AE maintained a large leaf area (> 78 cm²) in both rootstocks. On the other hand, under 3000 ppm, GB better preserved leaf number in Gahrawy (19.07 leaves), while AE better maintained leaf area in Sokkary (75.66 cm²) Table 1 and Fig. 2A and B. Also, results in the same Table 1; Fig. 2-C clarified that leaf thickness increased at moderate salinity (1500 ppm: 0.270 mm) and was enhanced by both treatments (~ 0.26 mm compared to 0.244 mm control). Sokkary consistently maintained thicker leaves across combinations, with AE under tap water producing the maximum thickness (0.312 mm), indicating structural adaptation to stress.

**Fig. 3:**
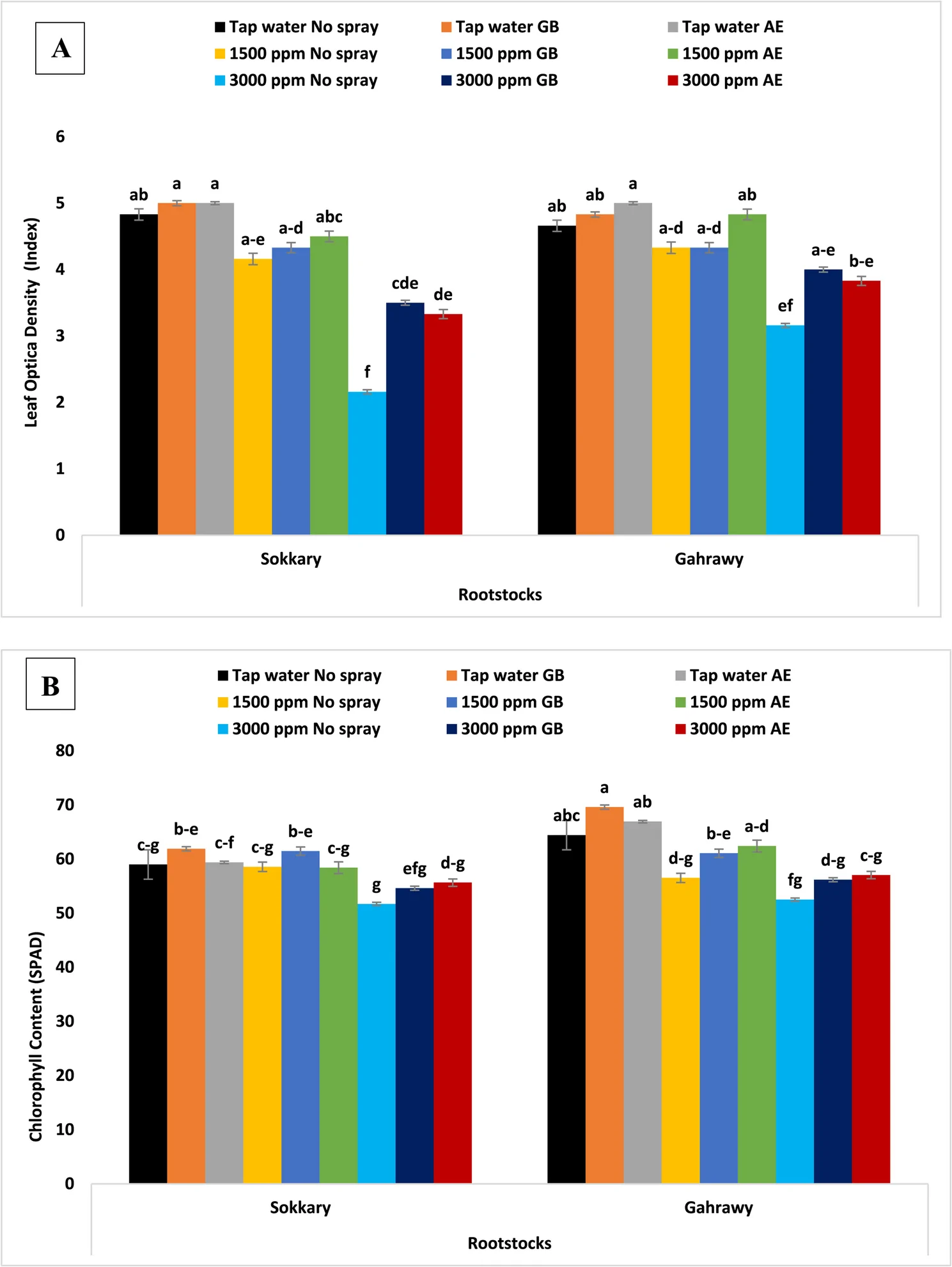
Interaction Effect of salinity levels and spraying treatments of glycine betaine (GB) and seaweed algae extracts AE on Leaf Optical Density )(**A**) and Chlorophyll content (SPAD) (**B**) of mango rootstock seedlings

Data presented in Table 1; Fig. 3-A indicated that leaf optical quality was significantly affected by salinity, rootstock genotype, biostimulant treatments, and their interactions. Salinity caused a progressive decline in optical quality, with the lowest value recorded in Sokkary at 3000 ppm under the unsprayed treatment (2.16), whereas both rootstocks maintained high values under tap water (4.66–5.00). Foliar application of GB and AE partially alleviated the negative effects of salinity, increasing optical quality at 3000 ppm to 3.50 and 3.33 in Sokkary and to 4.00 and 3.83 in Gahrawy, respectively. Under moderate salinity (1500 ppm), AE was slightly more effective than GB in Gahrawy (4.83 vs. 4.33). These responses indicate that Gahrawy had greater inherent tolerance, while biostimulants provided additional protection, particularly under severe salinity.

Regarding chlorophyll content (SPAD values) Table 1; Fig. 3-B, the interaction between rootstock, salinity, and biostimulant treatments demonstrated clear genotype-dependent responses. Under non-saline conditions, GB produced the highest chlorophyll content in Gahrawy (69.60). At moderate salinity (1500 ppm), GB was more effective in Sokkary, increasing SPAD to 61.47, whereas AE performed better with Gahrawy (62.42). Under severe salinity (3000 ppm), chlorophyll content declined across treatments, reflecting the stronger inhibitory effect of salt stress on photosynthetic pigments; however, AE in Gahrawy and GB in Sokkary retained relatively higher values. Overall, the results suggest that both biostimulants can help preserve leaf optical quality and chlorophyll under salinity, but their effectiveness depends strongly on rootstock genotype and stress intensity.

### Potassium (K%) and sodium (Na%) accumulation

**Fig. 4:**
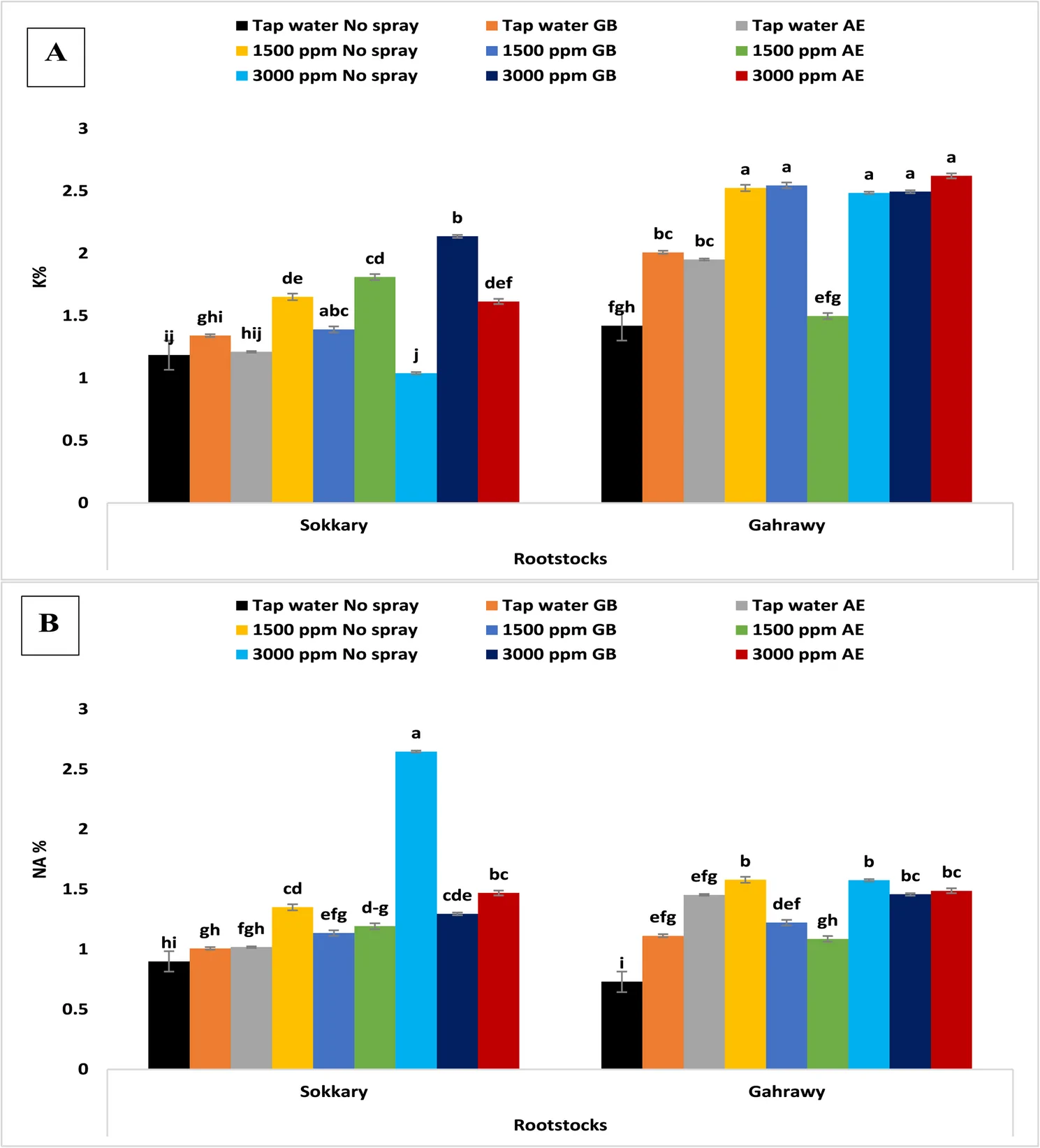
Interaction Effect of salinity levels and spraying treatments of glycine betaine (GB) and seaweed algae extracts AE on K (**A**) and Na (**B**) percentages of mango rootstock seedlings

The data presented in Table 1; Fig. 4-A indicated that Gahrawy maintained significantly higher leaf K% than Sokkary, with mean values of 2.174 and 1.488%, respectively, reflecting its greater capacity to maintain K⁺ homeostasis under saline conditions. Salinity progressively increased K% from 1.521% under tap water to 1.905 and 2.066% at 1500 and 3000 ppm, respectively, suggesting an adaptive response to salt stress. Among the anti-saline treatments, GB produced the highest mean K% (1.987%), followed by AE (1.786%) and the unsprayed control (1.720%). The interaction further confirmed the superiority of Gahrawy across treatment combinations, while GB was particularly effective under severe salinity, increasing K% to 2.138% in Sokkary and 2.495% in Gahrawy at 3000 ppm.

Conversely, Na% Fig. 4-B showed an opposite trend, with Sokkary accumulating more Na⁺ than Gahrawy (1.335 vs. 1.266%), indicating a less favorable ionic balance. Increasing salinity markedly enhanced Na% from 0.985% under tap water to 1.262% at 1500 ppm and 1.656% at 3000 ppm. Both GB and AE significantly reduced Na% compared with the unsprayed control (1.463%), reaching 1.234 and 1.206%, respectively. This reduction was more pronounced under moderate and severe salinity, particularly in Sokkary at 3000 ppm, where GB and AE reduced Na% from 2.647% in the unsprayed plants to 1.296 and 1.470%, respectively. These findings indicate that GB and AE can improve ionic balance by enhancing K⁺ retention and restricting Na⁺ accumulation, particularly under high salinity.

### Physiological and Biochemical Parameters

#### Antioxidant and osmoprotectant activity

**Fig. 5:**
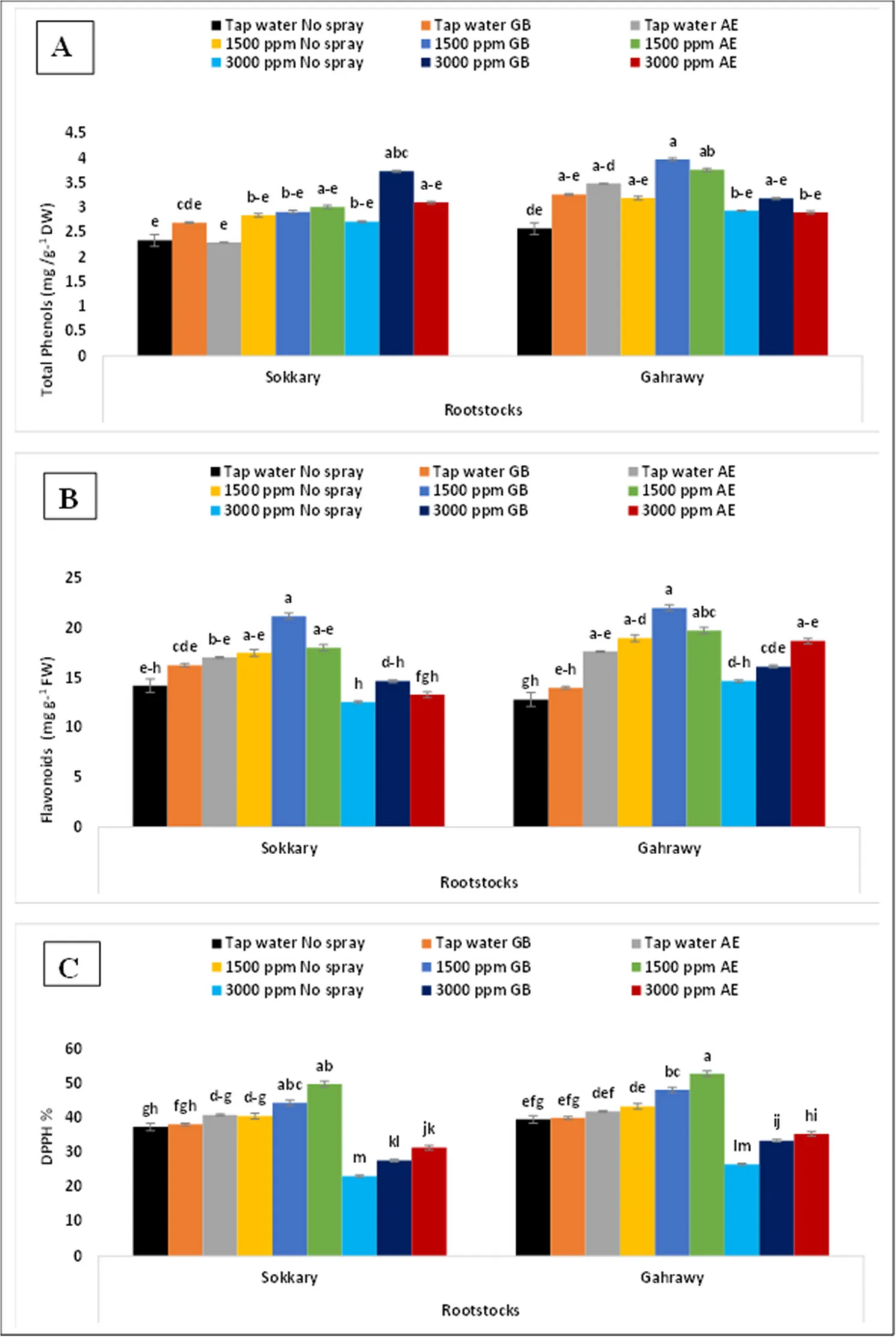
Interaction Effect of salinity levels and spraying treatments of glycine betaine (GB) and seaweed algae extracts AE on total phenols (mg g^− 1^ DW) (**A**) flavonoids (mg g^− 1^ FW) (**B**) and DPPH% (**C**) of mango rootstocks seedlings

Data presented in Table 2; Fig. 5-A showed that phenolic content increased under moderate salinity, reaching 3.29 mg g⁻¹ DW at 1500 ppm compared with 2.78 mg g⁻¹ DW under tap water, indicating stimulation of secondary metabolism by moderate salt stress. However, phenolic content slightly declined at 3000 ppm (3.10 mg g⁻¹ DW), suggesting that severe stress may limit the capacity for phenolic biosynthesis. Both GB and AE enhanced phenolic accumulation, although their effects varied with rootstock and salinity level. Under tap water, AE increased phenolic content in Gahrawy to 3.49 mg g⁻¹ DW, while at 1500 ppm, GB produced the highest value in Gahrawy (3.98 mg g⁻¹ DW). At 3000 ppm, GB markedly increased phenolics in Sokkary (3.74 mg g⁻¹ DW), demonstrating genotype-specific responses.

**Table 2:** Main effects of the investigated factors on mango rootstocks under salinity conditions as response of oxidative markers, enzymatic and non-enzymatic antioxidants characteristics

Main Factors	Flavonoids mg g^− 1^ FW	Total phenols mg g^− 1^ DW	Proline µmol g^− 1^ FW	DPPH %	MDA µmol g^− 1^ FW	H_2_O_2_ µmol mg^− 1^ FW	PPO U(µmol)/g FW	APX U(µmol)/g FW	POD U(µmol)/g FW	Glycine Betaine (µmol)/g DW
Rootstocks										
Sokkary	16.11	2.85b	25.93	37.13a	17.47	7.48b	49.65b	2.66a	36.00b	2.20b
Gahrawy	17.21a	3.26a	26.52	40.23b	18.28	10.85a	63.14a	1.99b	44.80a	2.91a
F ≤ 0.05	**ns**	*	**ns**	*	**ns**	*	*	*	*	*
Salinity Levels										
Tap Water	15.35b	2.78b	26.91	39.74b	16.11c	8.13b	37.49c	2.22b	24.30c	2.387b
1500 ppm	19.61a	3.29a	26.32	46.64a	17.52b	9.28a	59.60b	2.60a	33.48b	2.624a
3000 ppm	15.02b	3.10a	26.45	29.64b	20.00a	10.09a	72.10a	2.16b	64.53a	2.670a
F ≤ 0.05	*	*	**ns**	*	*	*	*	*	*	*
Anti-Saline										
Non	15.14b	2.77b	27.2a	35.17c	19.76a	10.76a	63.56a	2.43a	49.46a	2.287c
Glycine Betaine	17.40a	3.30a	25.60b	38.70b	16.97b	8.47b	54.56b	2.27b	41.84a	2.970a
Algae Extracts	17.49a	3.10a	25.90b	42.16a	16.90b	8.26b	51.07b	2.27b	31.02c	2.425b
F ≤ 0.05	*	*	**ns**	*	*	*	*	*	*	*

Note: * and ** indicate significant and highly significant effects, respectively

Flavonoid content (Fig. 5-B) was similarly influenced by salinity and biostimulant application, reaching its highest overall mean at 1500 ppm (19.61 mg g⁻¹ FW) compared with 15.35 mg g⁻¹ FW under tap water. GB was particularly effective under moderate salinity, producing 21.25 and 22.05 mg g⁻¹ FW in Sokkary and Gahrawy, respectively. Under severe salinity, flavonoid accumulation generally declined, although AE maintained a relatively higher level in Gahrawy (18.72 mg g⁻¹ FW), suggesting better preservation of antioxidant defenses under severe stress.

DPPH antioxidant activity (Table 2; Fig. 5-C) was significantly affected by rootstock, salinity, biostimulant treatment, and their interactions. Gahrawy showed a higher overall antioxidant activity than Sokkary (40.23 vs. 37.13%), indicating greater inherent antioxidant capacity. DPPH activity increased under moderate salinity, reaching 46.64% at 1500 ppm, but declined markedly to 29.64% at 3000 ppm, reflecting the damaging effects of severe salt stress. Both GB and AE improved DPPH activity, particularly under moderate salinity. AE produced the strongest response, reaching 50.00% in Sokkary and 53.00% in Gahrawy at 1500 ppm, while also partially improving activity at 3000 ppm. The highest value (53.00%) was therefore recorded in Gahrawy treated with AE at 1500 ppm, whereas the lowest (23.15%) occurred in untreated Sokkary under 3000 ppm. These results confirm that antioxidant response is strongly determined by the interaction between genotype, salinity severity, and biostimulant application. 

#### Osmoprotectants activity

**Fig. 6:**
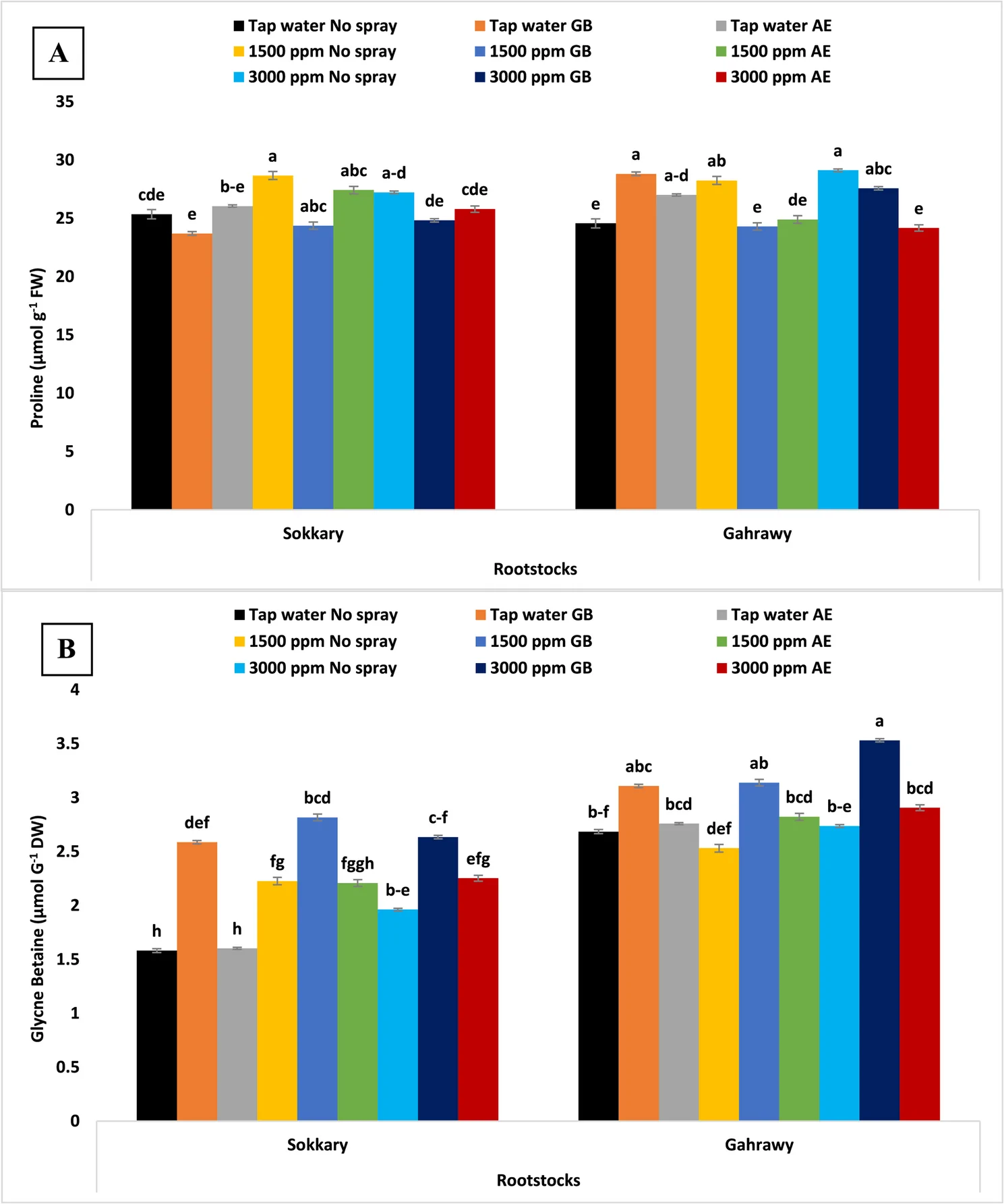
Interaction Effect of salinity levels and spraying treatments of glycine betaine (GB) and seaweed algae extracts AE on proline (µmol g^− 1^ FW) (**A**) and glycine betaine (µmol g^− 1^ DW) (**B**) of mango rootstocks seedlings

Respecting to the proline content of leaf rootstock, data in Table 2; Fig. 6-A showed no significant main effects of rootstock or salinity, but unsprayed plants accumulated higher levels (27.2 µmol g⁻¹ FW) than treated plants, indicating that GB and AE alleviated osmotic stress. Interaction analysis showed that under 1500 ppm, untreated plants accumulated the highest proline (> 28 µmol g⁻¹ FW), whereas treatments reduced accumulation. Under 3000 ppm, proline levels stabilized across spraying treatments, suggesting threshold osmotic adjustment.

Regarding glycine betaine (GB), clear genotypic and treatment-dependent differences were detected in Table 2; Fig. 6-B. Gahrawy rootstock showed a higher mean GB content than Sokkary (2.91 compared to 2.20 µmol g⁻¹ DW), indicating a greater intrinsic capacity to accumulate compatible solutes under the prevailing conditions. Salinity significantly promoted GB accumulation relative to tap water, with both 1500 and 3000 ppm yielding higher GB means (2.624 and 2.670 µmol g⁻¹ DW, respectively) than the control (2.387 µmol g⁻¹ DW). Foliar anti-saline treatments further enhanced GB, with glycine betaine spray giving the highest overall value (2.970 µmol g⁻¹ DW), followed by algae extract (2.425 µmol g⁻¹ DW), while the unsprayed control recorded the lowest GB (2.287 µmol g⁻¹ DW). Interaction data in (Fig. 5-D) revealed that under tap water, GB spray markedly increased leaf GB in both rootstocks compared with no sprayed seedlings, and with both 1500 and 3000 ppm salinity, the combination of Gahrawy rootstock and GB spray generally produced the highest GB values, demonstrating that exogenous GB amplifies the stress-induced accumulation of this osmolyte in a genotype-dependent manner.

#### Oxidative stress markers accumulations

**Fig. 7:**
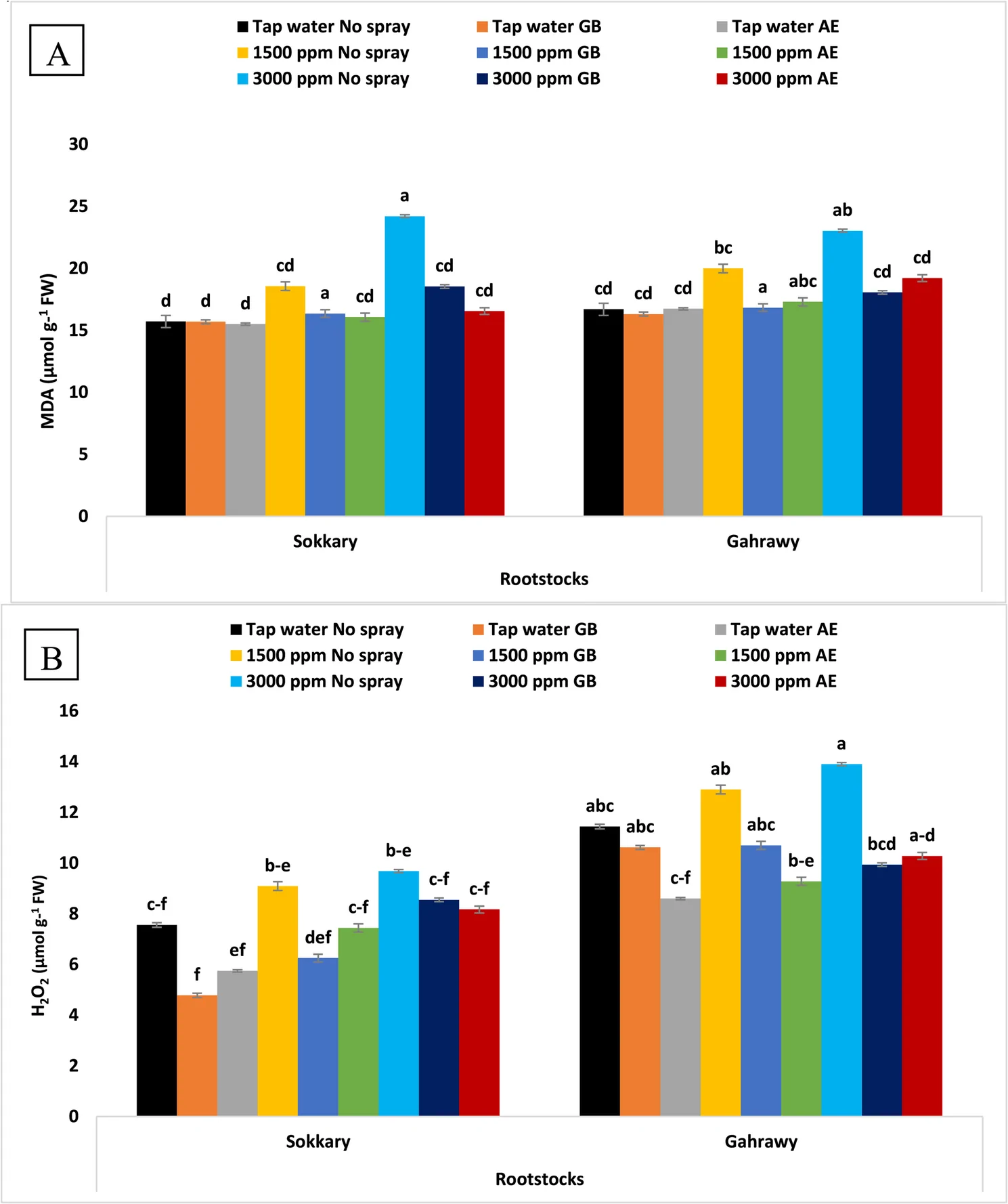
Interaction Effect of salinity levels and spraying treatments of glycine betaine (GB) and seaweed algae extracts AE on MDA (µmol g-1 FW) (**A**) and H2O2 (µmol g-1 FW) of mango rootstocks seedlings (**B**)

Regarding to oxidative markers and their accumulation illustrated data in Table 2  and (Fig.7-A) revealed that, Malondialdehyde (MDA) increased progressively with salinity (16.11 to 20.00 µmol g⁻¹ FW), while GB and AE significantly reduced lipid peroxidation (approximately 16.9 compared to 19.76 control). Interactive responses showed minimal differences under tap water. Whereas, at 1500 ppm, antistress treatments GB and AE suppressed MDA accumulation, especially in Sokkary. Under 3000 ppm, untreated plants showed severe membrane damage (up to 24.21 µmol g⁻¹ FW), while spraying treatments GB and AE reduced MDA to 16–19 µmol g⁻¹ FW, confirming their role in membrane protection. As for hydrogen peroxide accumulation, increasing salinity increased H₂O₂ levels, while GB and AE significantly reduced accumulation. Also, related to rootstock effects, Gahrawy rootstock accumulated significantly more H₂O₂ than Sokkary. Across interactions (Fig. 7-B) presented that, Sokkary consistently showed better ROS regulation. GB and AE effectively reduced H₂O₂ overall salinity levels. With increasing salinity, treatments provided greater ROS reduction in Sokkary than Gahrawy, indicating genotypic differences in oxidative metabolism.

### Enzymes activity

**Fig. 8:**
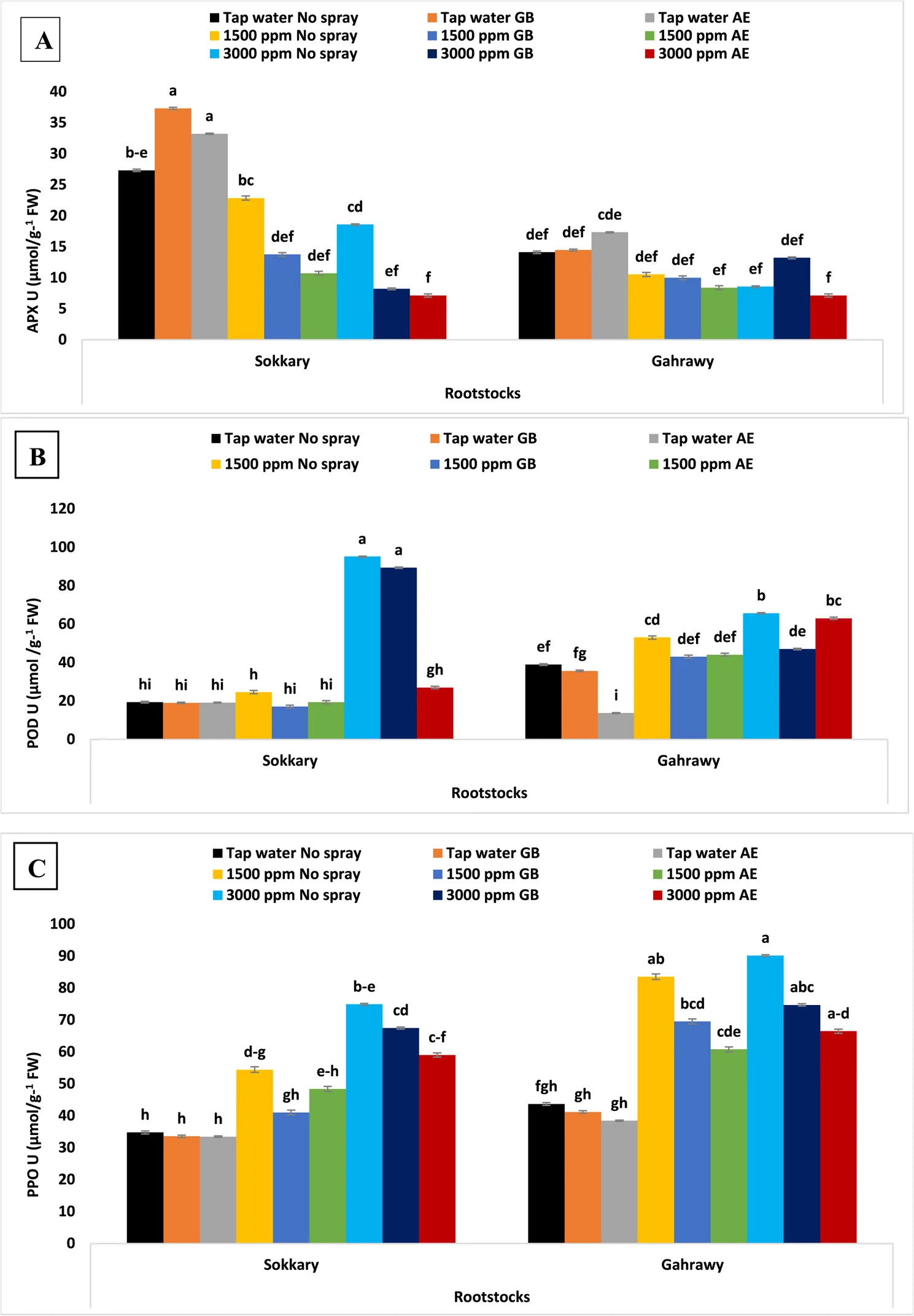
interaction effect of salinity levels and spraying treatments of glycine betaine (GB) and seaweed algae extracts AE on APX U(µmol g^− 1^ FW) (**A**), POD U(µmol g^− 1^ FW) (**B**) and PPO U(µmol g^− 1^ FW) (**C**) enzymes activity of mango rootstocks seedlings

The activities of key antioxidant enzymes revealed significant responses to all investigated factors Table 2; Fig. 8A, B and C. Ascorbate peroxidase APX activity was higher in Sokkary and at moderate salinity, then declined at 3000 ppm. Control plants exhibited higher activity than treated plants. APX activity was significantly higher in Sokkary (2.66 U (µmol g^− 1^ FW) than Gahrawy (1.99 U(µmol g^− 1^ FW) ). The APX activity peaked at 1500 ppm (2.60 U g⁻¹ FW), followed by a decline at 3000 ppm (2.16 U (µmol g^− 1^ FW). As for the interaction effect, APX activity demonstrated declining patterns under increasing stress intensity. Under non-saline conditions, Sokkary maintained higher APX activity than Gahrawy across all treatments (27.32–37.32 vs. 14.10-17.32 U(µmol g^− 1^ FW)). At 1500 ppm salinity, APX activity decreased substantially in both rootstocks, with more pronounced reductions in treated plants compared to controls. Under severe salinity (3000 ppm), APX activity further declined across all treatment combinations, with values ranging from 7.14 to 18.57 U(µmol g^− 1^ FW), suggesting possible enzyme degradation or feedback inhibition under prolonged severe stress. The general pattern indicated that while Sokkary maintained higher basal APX activity, both rootstocks experienced significant enzyme downregulation under salinity stress, with anti-saline treatments unable to prevent this decline, possibly reflecting a shift toward other antioxidant defense mechanisms under severe stress conditions, suggesting enzyme downregulation under prolonged stress. Also, data presented in Table 2 and (Fig. 8-B) demonstrated thatperoxidase activity (POD) in mango rootstocks was significantly influenced by rootstock, salinity level, and foliar application of anti-saline compounds, as well as by their interactions. Gahrawy exhibited a higher mean POD (44.8 U µmol g⁻¹ FW) than Sokkary (36 U µmol g⁻¹ FW), indicating a stronger oxidative response in this rootstock under the tested conditions. Increasing salinity markedly enhanced POD, from 24.30 under tap water to 33.48 at 1500 ppm and 64.53 at 3000 ppm, confirming induction of oxidative stress by salt exposure. As for interaction effects across factors, non-sprayed plants showed the highest POD (49.46), whereas glycine betaine (GB) and algae extract (AE) reduced POD to 41.84 and 31.02, respectively, suggesting that both treatments mitigated oxidative stress, with a stronger overall effect of AE. However, interaction data Fig. 8-B. revealed that the magnitude of this mitigation was strongly dependent on rootstock and salinity level. In Sokkary, AE was particularly effective at 3000 ppm, reducing POD from 95.1 (non-sprayed) to 27, whereas GB produced only a modest decrease (89.36). Consistent with that with Gahrawy, GB more consistently lowered POD at moderate and high salinity ( from 65.66 to 47 at 3000 ppm), whereas AE produced a pronounced high reduction under non-saline conditions (from 39.0 to 13.66 under tap water). Collectively, these results indicate that while increasing salinity universally elevates POD and both GB and AE reduce oxidative stress. Main effects as for polyphenol oxidase (PPO) activity in Table 2 exhibited contrasting rootstock responses, with Gahrawy (63.14 U(µmol)g-^1^ FW) showing significantly higher activity than Sokkary (49.65 U(µmol)/g FW). PPO activity increased progressively with salinity stress, from 37.49 U(µmol)/g FW with (tap water) unsprayed seedlings to 59.60 U(µmol)g-^1^ FW (1500 ppm) and 72.10 U(µmol)/g FW (3000 ppm). Regarding to interactive pattern Fig. 8-C (PPO) activity illustrated complex enzyme regulation under stress (Fig. 6-B). Across all salinity levels, Gahrawy consistently exhibited higher PPO activity than Sokkary. Under tap water conditions, differences were moderate (Gahrawy: 33.42–43.66 compared to Sokkary: 33.42–34.75 U(µmol g^− 1^ FW). At moderate salinity (1500 ppm), untreated Gahrawy showed dramatic PPO induction (83.53 U/ (µmol g-^1^ FW), while anti-saline treatments substantially moderated this activation (GB: 59.50, AE: 60.77 U(µmol g-^1^ FW). Under severe stress (3000 ppm), un sprayed plants of both rootstocks exhibited maximum PPO activity (Sokkary: 74.87, Gahrawy: 90.14 U/( µmol g-^1^ FW), suggesting that anti-saline treatments reduced oxidative stress requiring PPO activation.

**Fig. 9:**
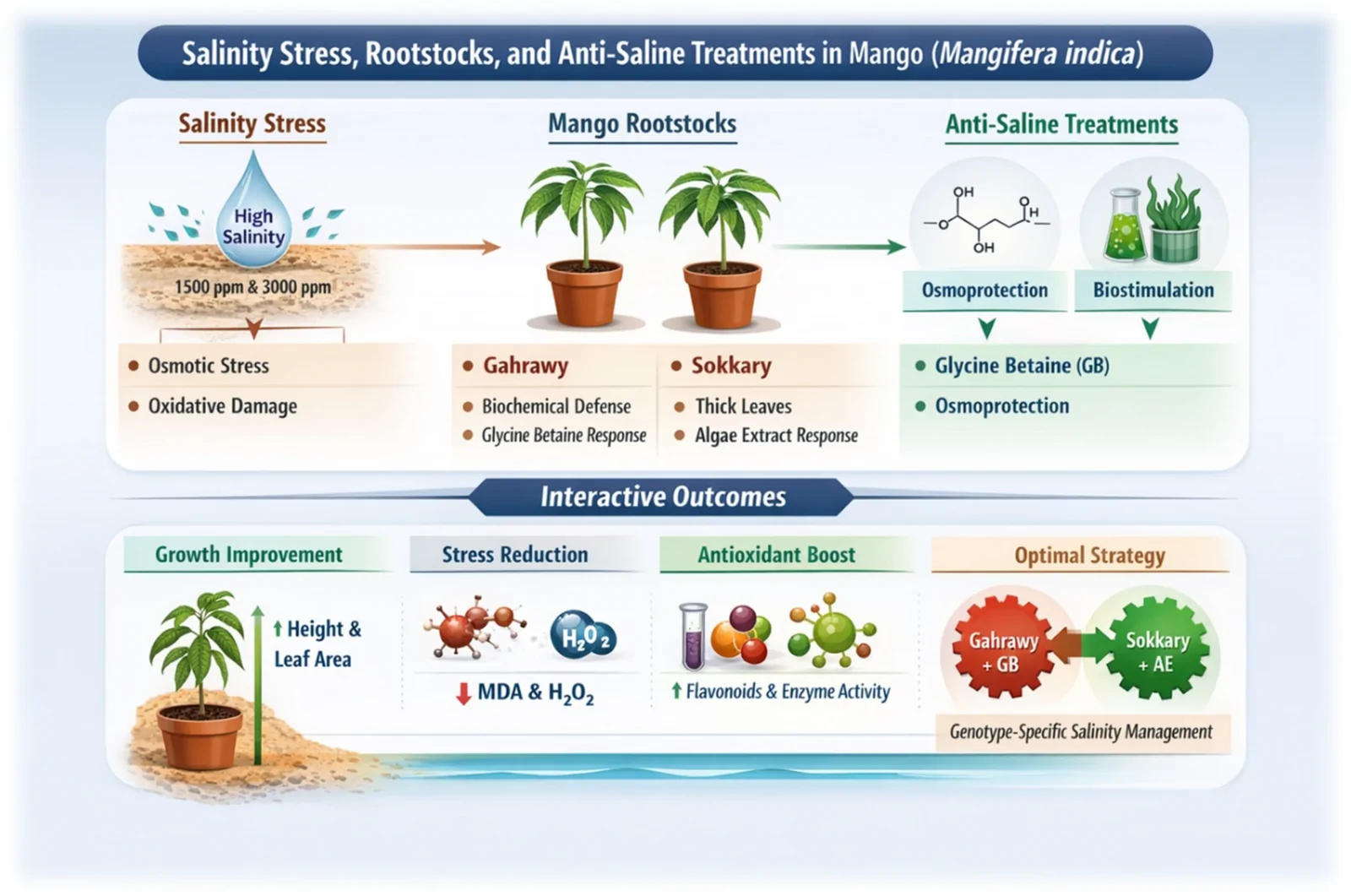
Descriptive diagram of interaction patterns of salinity levels, rootstock type and antistress biostimulants AE and GB on mango rootstocks

The interaction patterns among salinity levels, rootstock types, and antistress biostimulants (AE and GB) are illustrated in Fig. 9, showing the combined effects of these factors on the evaluated responses.

### Anatomical description of mango rootstock leaf texture

**Fig. 10:**
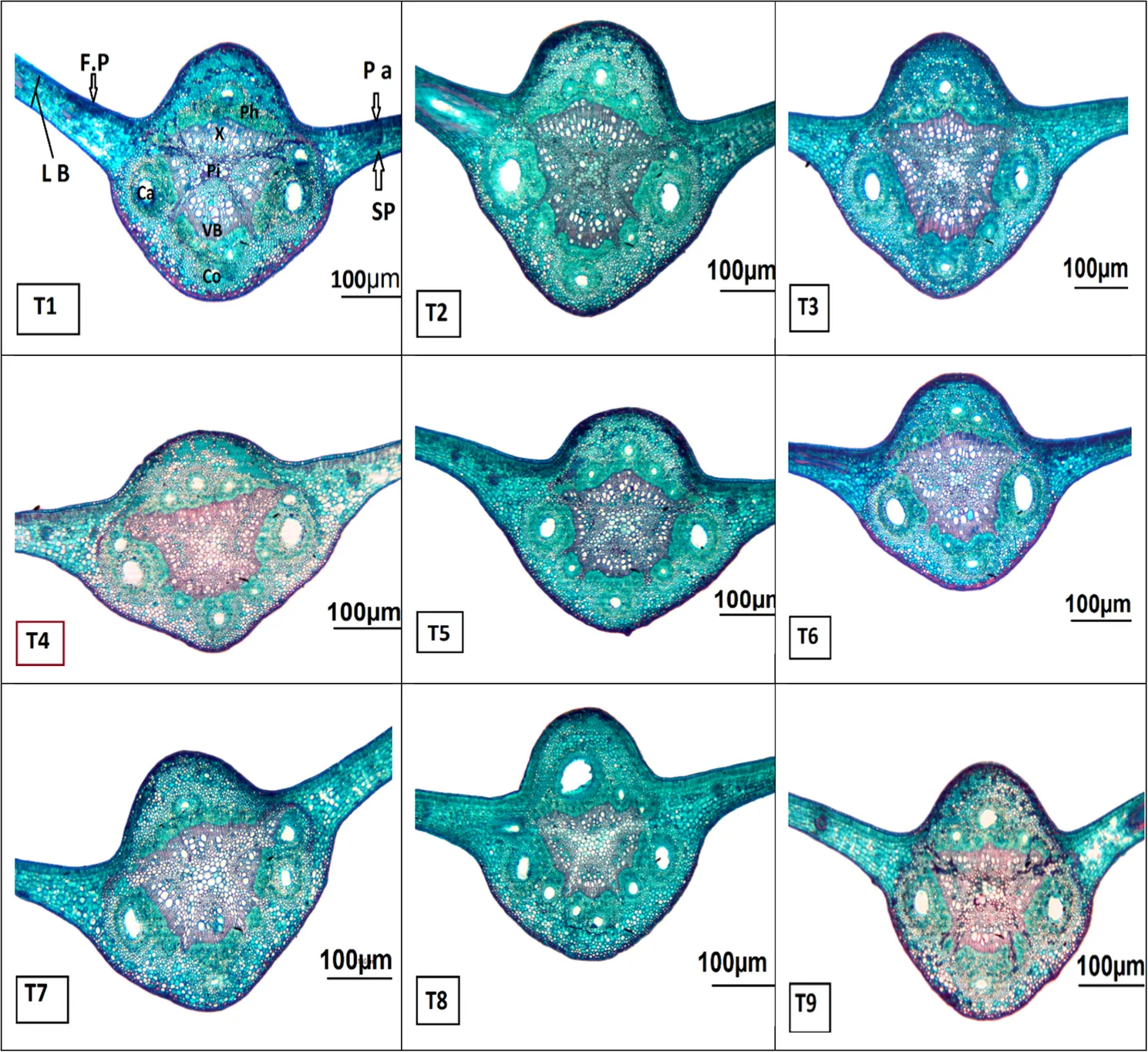
The transverse section through the leaves blade on the median portion of Sokkary mango rootstock as affected by salinity stress and anti stress (GB and AE) shows asymmetric. (T1) Control with tap water without anti stress (T2) tap water with GB (T3) tap water with AE (T4) 1500 ppm alone, (T5) 1500 ppm + GB; (T6) 1500 ppm + AE; (T7) 3000 ppm alone; (T8) 3000 ppm + GB and (T9) 3000 ppm + AE; Ep: epidermal Pa: palisade tissue; Ca: Secretory duct; SP: spongy tissue, UE: upper epidermis, VB: vascular bundle, X: xylem, Ph: phloem, Co: cortex, LB: leaf blade

Nine leaf samples per cultivar of *Mangifera indica* L. were subjected to quantitative anatomical analysis across treatments comprising three salinity levels (0, 1500, and 3000 ppm) and two foliar biostimulants — glycine betaine (GB) and algae extract (AE) — relative to an untreated tap-water control. In the Sokkary rootstock Table 3; Fig. 10, cuticle thickness increased under 1500 ppm salinity alone (3.1 μm vs. 2.5 μm in the control) but decreased to 2.1 μm when GB was co-applied at either salinity level, suggesting that GB alleviates the hydraulic stress that drives cuticular deposition. Epidermal thickness peaked under 1500 ppm + GB (16.0 μm) and reached its minimum under 3000 ppm + GB (11.8 μm), indicating a concentration-dependent, potentially antagonistic interaction under severe salinity. Palisade and spongy mesophyll responded inversely: the 3000 ppm + GB combination promoted a compact, palisade-dominated architecture (48.5 and 91.7 μm, respectively; palisade: spongy ratio = 53%), whereas 3000 ppm salinity alone induced pronounced spongy tissue hypertrophy (145.1 μm; ratio = 27%), implying that unchecked severe salinity disrupts mesophyll organisation. Total lamina thickness followed a parallel trend, peaking at 199.6 μm under 3000 ppm only and declining to a minimum of 145.9 μm under 1500 ppm + AE. Midrib width was greatest under 3000 ppm salinity alone (1,554 μm) and equivalent in the control and 3000 ppm + GB treatments (~ 1,250 μm), while the largest vascular bundles (453.8 × 510.1 μm), thickest xylem (304.9 μm) and phloem (105.5 μm), and widest xylem vessels (37.7 μm) were all recorded under 3000 ppm only, collectively indicating a compensatory expansion of the vascular system to sustain water and solute transport under severe ionic stress. Maximum pith height (230.5 μm) and cortex thickness (238.6 μm) also occurred under 3000 ppm without biostimulant, whereas the greatest pith width was observed under 3000 ppm + AE (311.0 μm). Sub-epidermal sclerenchyma was most developed under 1500 ppm + AE (38.4 μm adaxially; 33.2 μm abaxially), suggesting that AE selectively reinforces mechanical support tissue at moderate salinity. Finally, secretory ducts attained their largest dimensions under 3000 ppm + GB (height 125.1 μm; width 113.2 μm), potentially reflecting enhanced biosynthesis of stress-related secondary metabolites when severe salinity is partially mitigated by GB.

**Table 3:** leaves anatomy characteristics of Sokkary cultivar under salinity stress with GB and AE applications:

Measured traits (µm)	T1	T2	T3	T4	T5	T6	T7	T8	T9	F. Test (*P* < 0.05).
Cuticle layer	2.5a-d	3ab	2.7abc	3.1a	2.1 cd	2.3bcd	2.8abc	2.1 cd	1.9d	**
Epidermal layer	12de	12.5de	11.9de	12.60de	16.0a	13 cd	14.7b	11.8e	13.7bc	**
Palisade	40.6d	41.7c	35.9i	47.40b	48.5a	37.9 g	39.1f	39.8e	37.2 h	**
Spongy	108.5b	91.5f	97.9d	93.80e	91.7f	88.7 g	145.1a	88.3 g	103.0c	**
P/S	0.37	0.45	o.36	0.51	0.53	0.43	0.27	0.45	0.36	ns
midrib region length	1252.0d	1432.0a	1313.0b	1177.0 h	1242.0e	1174.0i	1264.0c	1201.5 g	1212.6f	**
midrib region – width	1250.0i	1514.0b	1484.0d	1502.0c	1427.0e	1344.0 g	1545a	1401.5f	1252.0 h	**
Leaf-blade	171.9c	157.3e	148.2 g	173.1b	167.2d	145.6 h	199.6a	149.6f	157.1e	**
Xylem	222.9e	272.3b	243.2c	166.9i	212.4f	224.0d	304.9a	174.4 h	197.9 g	**
Phloem	104.2b	82.0 g	91.1e	89.4f	81.6 g	93.3d	105.5a	105.3a	95.1c	**
Vascular Bundle -length	400.7c	325.6e	317.5 g	406.5b	281.6 h	324.2f	453.8a	247.4i	341.6d	**
Vascular bundle - width	379.8d	485.9c	292.1 g	508.6b	341.1	336.3f	510.1a	287.9 h	341.1e	**
Xylem vessel-diameter	32.4e	32.9d	37.4b	30.2f	28.1 g	35.3c	38.7a	21.9 h	33.2d	**
Pith-length	101.0f	101.0f	142.6e	188.4d	208.4c	66.3 h	230.5a	90.5 g	229.6b	**
Pith- width	177.9e	107.4 h	181.6c	180.5d	143.2 g	102.6i	172.6f	205.3b	311.0a	**
Cortex	167.5i	220.8e	177.1 h	203.7 g	236.6b	208.6f	238.7a	232.4c	225.8d	
Sclerenchyma (upper)	30.8b	27.5c	22.4f	18.9 g	26.1d	38.4a	24.9e	18.9 g	22.5f	**
Sclerenchyma (lower)	32.6b	29.8c	19.6 g	22.1f	27.2d	33.2a	22.4f	25.4e	29.8c	**
Canal-length	109.5e	129.0a	106.3f	117.5d	97.4 g	73.2i	91.6 h	125.8b	119.5c	**
Canal-width	87.0e	112.4a	83.3f	98.4c	112.7a	101.0b	75.6 g	113.2a	96.1d	**

(T1) Control with tap water without anti stress (T2) tap water with GB (T3) tap water with AE (T4) 1500 ppm alone, (T5) 1500 ppm + GB; (T6) 1500 ppm + AE; (T7) 3000 ppm alone; (T8) 3000 ppm + GB and (T9) 3000 ppm + AE

Note: * and ** indicate significant and highly significant effects, respectively

**Fig. 11:**
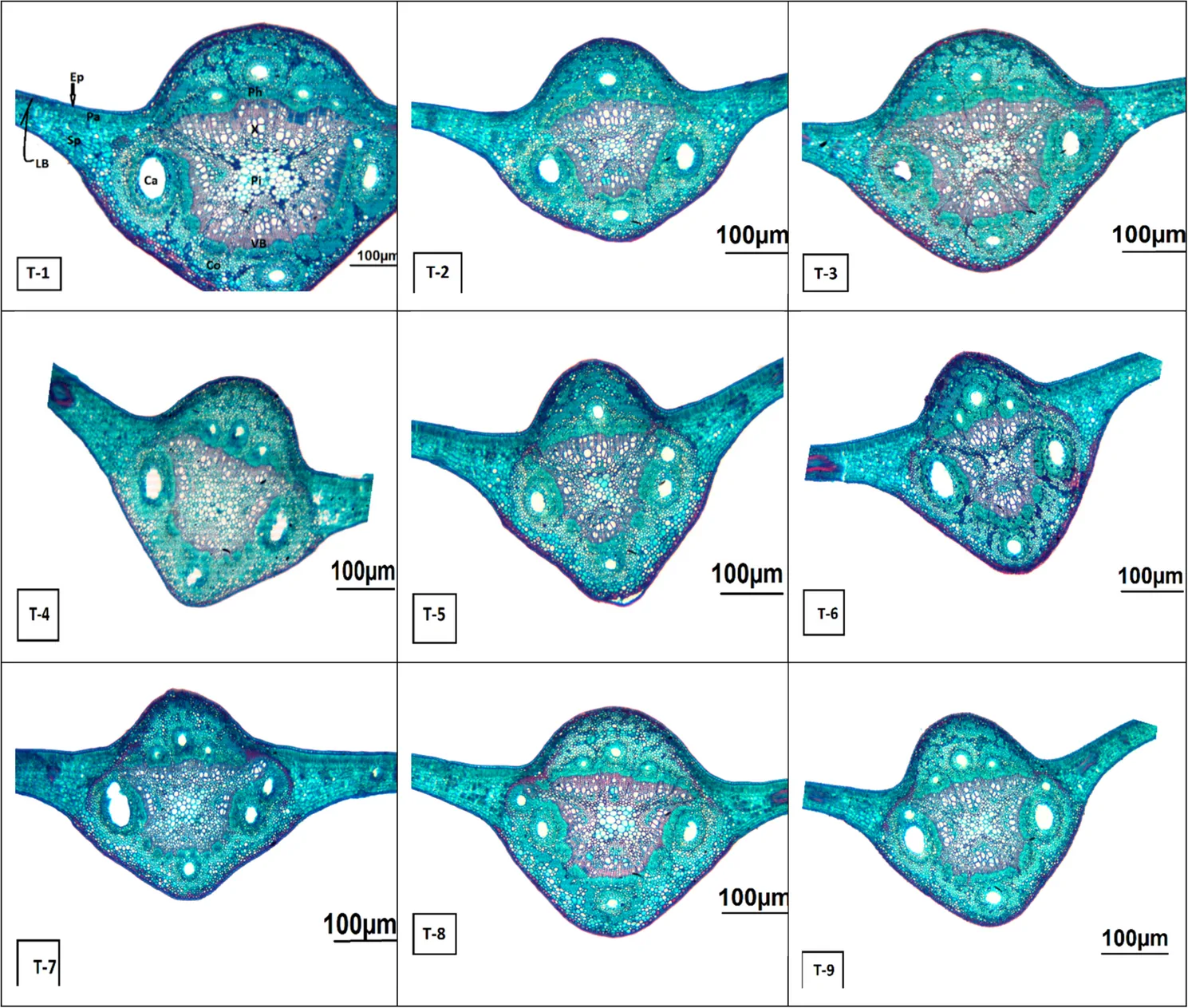
The transverse section through the leaves blade on the median portion of Gahrawy mango rootstock as affected by salinity stress and anti stress (GB and AE) shows asymmetric. (T1) Control with tap water without anti stress (T2) tap water with GB (T3) tap water with AE (T4) 1500 ppm alone, (T5) 1500 ppm + GB; (T6) 1500 ppm + AE; (T7) 3000 ppm alone; (T8) 3000 ppm + GB and (T9) 3000 ppm + AE; Ep: epidermal Pa: palisade tissue; Ca: Secretory duct; SP: spongy tissue, UE: upper epidermis, VB: vascular bundle, X: xylem, Ph: phloem, Co: cortex, LB: leaf blade

The Gahrawy rootstock displayed a broadly similar anatomical response to Sokkary, yet with notable genotype-specific shifts reflecting differential anatomical plasticity under combined salinity and biostimulant treatments Tables 4 and Fig. 11. Cuticle thickness peaked under 1500 ppm + GB (4.0 vs. 3.3 μm in the control) while epidermal thickness was simultaneously minimized, implying a structural trade-off between cuticle deposition and epidermal cell expansion; conversely, maximum epidermal thickness (15.3 μm) occurred under 3000 ppm alone, confirming that severe ionic stress without biostimulant application promotes epidermal hypertrophy. Palisade tissue was thickest under 1500 ppm + GB (46.4 μm), whereas spongy mesophyll peaked under 3000 ppm only (106.4 μm), reducing the palisade: spongy ratio from 46% in the control to 39%, mirroring the pattern observed in Sokkary. Total lamina thickness reached a maximum of 167.5 μm under 3000 ppm only and a minimum of 122.5 μm under 3000 ppm + AE (control: 140.8 μm), while midrib dimensions were greatest under 3000 ppm only (height 1,464.7 μm; width 1,607.9 μm) yet markedly reduced by biostimulant co-application (990 × 1,315 μm), underscoring a decoupling between laminar and vascular axis growth responses. Unlike Sokkary, vascular bundle development in Gahrawy was most enhanced under tap water + AE (310.8 × 331.4 μm; xylem 228.7 μm; phloem 89.8 μm), whereas 3000 ppm salinity alone produced the smallest bundles (191.7 × 203.3 μm) with severely reduced xylem (154.3 μm) and phloem (63.5 μm), indicating that high salinity without biostimulant supplementation constrains vascular development in this genotype. GB at elevated salinity consistently promoted pith and cortex enlargement — maximum pith length (173.1 μm) and cortex thickness (219.3 μm) were both recorded under 3000 ppm + GB, contrasting with the smallest cortex under 3000 ppm alone (122.9 μm). Sub-epidermal sclerenchyma was most developed under 1500 ppm + AE (44.5 μm adaxially; 34.7 μm abaxially) and minimal under 3000 ppm only (16.1 μm). Secretory duct size was greatest under 3000 ppm treatments irrespective of biostimulant type (height 135.3 μm under 3000 ppm + GB; width 135.2 μm under 3000 ppm + AE) and minimised under tap water + AE (height 102.1 μm; width 85.5 μm), suggesting that secretory tissue enlargement is contingent on elevated salinity stress rather than on the specific biostimulant applied. 

**Table 4:** Leaves anatomy characteristics of Gahrawy rootstock under salinity stress with GB and AE applications

Measured traits ( µm)	T1	T2	T3	T4	T5	T6	T7	T8	T9	F. Test (*P* < 0.05).
Cuticle layer	3.3	3.4	3.5	4.0	4.0	3.0	3.5	3.8	3.4	ns
Epidermal layer	13.7cde	13.5de	14.6b	14.4bc	13.1e	14.0bcd	15.3a	13.7	13.7cde	**
Palisade	38.5e	36.6f	34.5 g	40.9d	46.4a	42.7c	42.4c	45.7b	32.8 h	**
Spongy	83.4 g	86.8f	96.8c	90.3e	95.8d	80.9 h	106.9a	97.4b	65.7i	**
P/S	0.46	0.31	0.36	0.45	0.50	0.53	0.39	0.47	0.50	ns
Midrib region length	1312.6b	1125.8 h	1364.7a	1165.7 g	1216.0e	1251.0d	990i	1292c	1206.0f	**
Midrib region width	1545c	1447f	1607.9a	1462e	1339 h	1550b	1315i	1496d	1429 g	**
Leaf blade	140.8 g	133.2 h	154.1d	150.1e	161.7c	146.9f	167.5a	165.8b	122.5i	**
Xylem	214.2c	205.7e	228.7a	155.5 h	190.8 g	223.9b	154.3i	200.3f	211.9d	**
Phloem	72.3d	67.8e	89.8a	65.2f	86.1b	89.8a	63.5 h	82.0c	64.2 g	**
Vascular Bundle length	309.5b	260.8 g	310.8a	224.9 h	268.2f	278.2c	191.7i	274.5e	274.9d	**
Vascular bundle width	307.1b	300.8c	331.4a	238.2 g	226.3 h	252.8f	203.3i	260.8d	260.3e	**
Xylem vessel diameter	34.7c	36.1b	38.9a	30.7e	38.9a	29.6f	23.2 h	27.4 g	33.5d	**
Pith length	149.9d	88.4i	143.2e	160.5b	112.1f	102.6 g	156.3c	173.1a	98.4 h	**
Pith width	232.0b	140.0f	206.8d	262.1a	112.1 h	133.1 g	166.8e	215.3c	140.0f	**
Cortex	242.7a	181.7e	166.3f	163.2 g	195.1d	197.0c	122.9i	219.3b	158.1 h	**
Sclerenchyma (upper)	20.8d	17.7e	22.8b	16.6f	21.9c	44.5a	16.1 g	20.5d	17.4e	**
Sclerenchyma (lower)	34.4b	17.7 h	40.7a	29.5e	34.7b	32.9c	32.1d	20.2 g	23.7f	**
Canal-length	112.6f	126.3d	102.1 g	116.5e	128.0c	135.3b	93.7 h	74.4i	151.1a	**
Canal-width	98.9d	103.8c	89.5e	104.0c	88.1f	109.3b	88.1f	71.4 g	135.2a	**

(T1) Control with tap water without anti stress (T2) tap water with GB (T3) tap water with AE (T4) 1500 ppm alone, (T5) 1500 ppm + GB; (T6) 1500 ppm + AE; (T7) 3000 ppm alone; (T8) 3000 ppm + GB and (T9) 3000 ppm + AE

Note: * and ** indicate significant and highly significant effects, respectively

### Pearson correlation between rootstock type, salinity levels and biostimulants antistress:

**Fig. 12:**
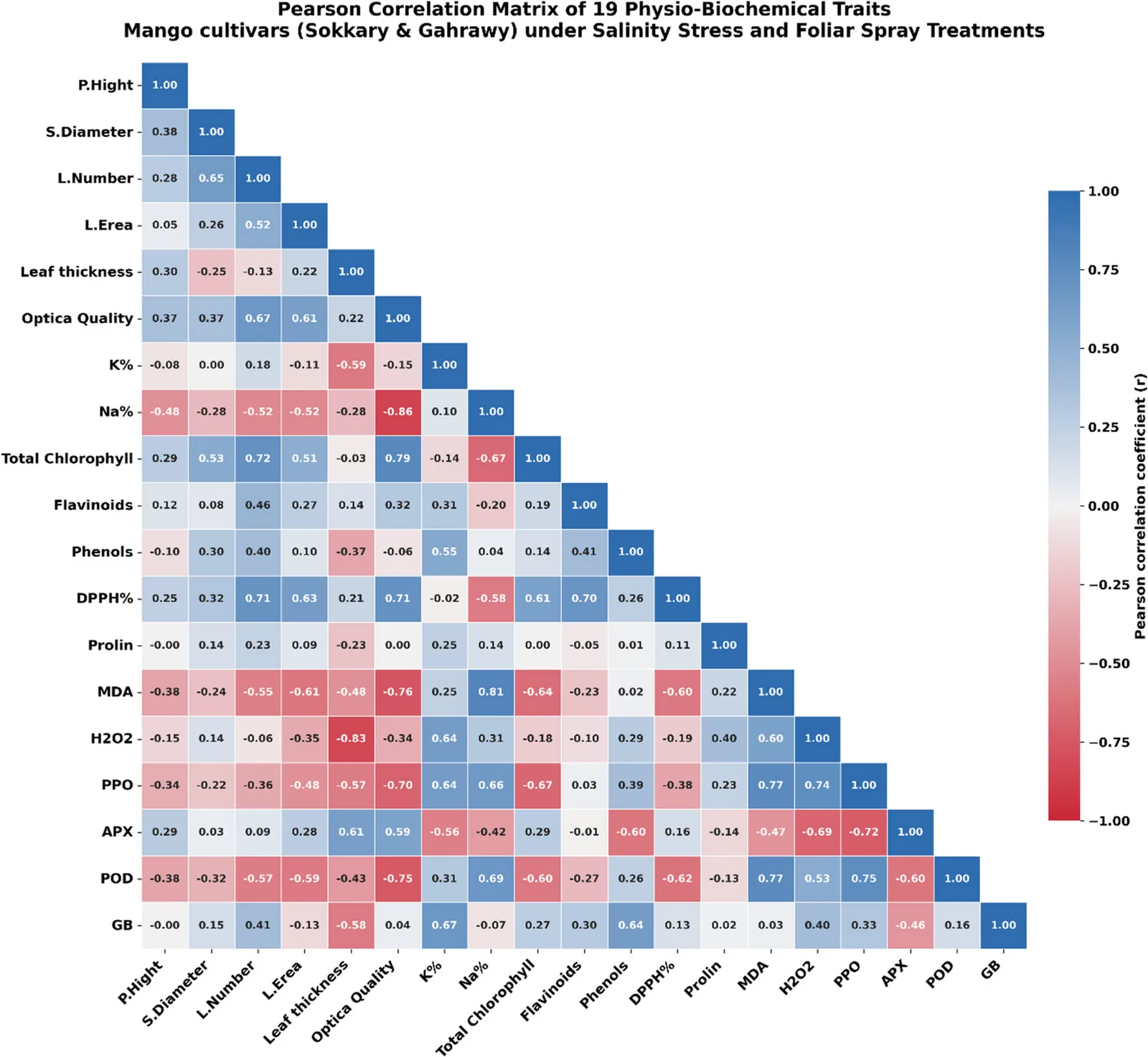
Pearson correlation heatmap showing relationships among growth parameters, physiological and biochemical attributes of mango rootstocks under salinity levels interactive with biostimulants GB and AE. Values within cells represent correlation coefficients (r), Red = strong positive correlation (close to + 1), Green = strong negative correlation (close to − 1)

Pearson correlation analysis Fig. 12 revealed a highly structured physiological network describing plant responses to salinity stress and the mitigating effects of foliar antistress biostimulants. Sodium accumulation (Na%) exhibited strong positive correlations with oxidative stress indicators such as MDA, H₂O₂, PPO, and POD, confirming that increased ionic toxicity promotes lipid peroxidation and activation of antioxidant enzymes. In contrast, Na% showed strong negative associations with optical quality, total chlorophyll, and several growth-related traits, indicating that salt-induced ionic imbalance directly suppresses photosynthetic capacity and plant vigor. Conversely, growth and physiological performance traits—including optical quality, leaf number, chlorophyll content, and DPPH scavenging activity—were positively correlated, suggesting that improved growth is closely linked with enhanced antioxidant capacity. These relationships also highlight the beneficial role of foliar-applied biostimulants, particularly glycine betaine and algae extract, which were associated with stronger antioxidant activity and improved physiological performance, likely through osmotic adjustment, stabilization of cellular structures, and activation of protective metabolic pathways under saline conditions.

**Fig. 13:**
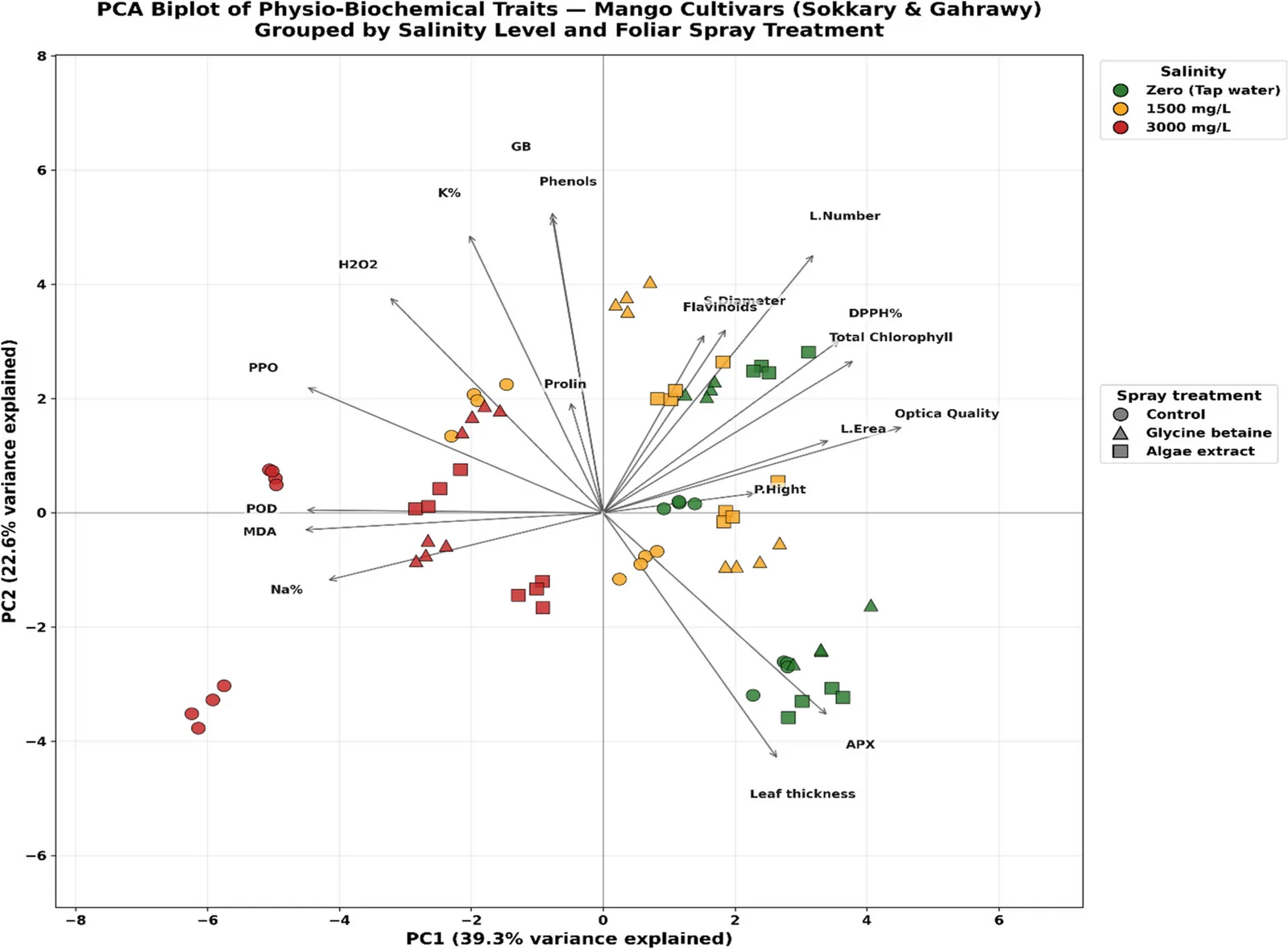
Principal component analysis (PCA) biplot among growth parameters, physiological and biochemical attributes of mango rootstocks under salinity levels interactive with biostimulants GB and AE

Principal Component Analysis Fig. 13 effectively summarized the multivariate dataset and revealed the dominant physiological gradients governing plant responses to salinity and biostimulant treatments. The first principal component (PC1) accounted for the largest proportion of total variance and represented the primary salinity stress–damage axis, contrasting traits associated with plant growth, chlorophyll content, and antioxidant capacity against stress-related traits such as Na%, MDA, and oxidative enzyme activities. This component clearly distinguished well-performing plants from severely salt-stressed individuals. The second principal component (PC2) captured an independent adaptive response axis characterized by the accumulation of osmoprotectants and secondary metabolites, including glycine betaine, phenolics, and potassium. The positioning of samples along this axis suggests that foliar application of glycine betaine and algae extract stimulated biochemical defense pathways and osmotic regulation mechanisms that contribute to enhanced salinity tolerance. Together, the PCA results demonstrate that plant responses to salinity are primarily organized along two complementary dimensions: the direct physiological damage caused by salt stress and the adaptive metabolic responses promoted by antistress biostimulant treatments.

## Discussion

The present study investigated the physiological, biochemical, and anatomical responses of ‘Sokkary’ and ‘Gahrawy’ mango rootstocks to salinity stress and the potential ameliorative roles of Glycine Betaine (GB) and Algae Extract (AE). Results demonstrated that salinity levels, particularly at 1500 and 3000 ppm, significantly impaired growth and induced oxidative and ionic stress across both genotypes. The application of GB and AE offered substantial and multi-faceted protection, with genotype-specific differences observed in the magnitude and mechanisms of tolerance. Plant height and stem diameter are reliable indicators of overall growth performance under abiotic stress [4, 8]. In the present study, salinity significantly reduced plant height, particularly at 3000 ppm, with untreated Sokkary and Gahrawy seedlings reaching only 24.00 and 25.25 cm, respectively, compared to over 40 cm under tap water. Specifically, plant height was observed at 43.70 cm at the 1500 ppm salinity level, reflecting clear growth retardation compared to non-stressed controls. These findings are consistent with those of *Semida et al. (2016)* [38], who reported that NaCl-induced salinity suppressed shoot elongation in vegetable crops by reducing turgor pressure and disrupting cell expansion. This reduction in growth parameters under salinity is attributed to the suppression of cell division and elongation, as well as the diversion of energy from growth to maintenance and stress adaptation mechanisms [4, 39]. The inhibitory effect of salt on plant height is primarily attributed to osmotic stress, which reduces water uptake, and ion toxicity caused by excessive Na⁺ and Cl⁻ accumulation in leaf tissues [40, 41].

Importantly, foliar application of Glycine Betaine (GB) substantially mitigated height reduction, particularly in Gahrawy at 1500 ppm, which achieved a maximum height of 50.00 cm, a 35% gain over untreated plants. GB acts as a compatible solute that maintains cell turgor and protects metabolic processes from the dehydrating effects of salinity [4]. This protective role of GB aligns with the work of *Annunziata et al. (2019)* [42], who demonstrated that exogenous GB stabilizes membrane integrity and promotes osmotic adjustment in salt-stressed plants by accumulating in the cytosol and chloroplasts without interfering with normal metabolic processes. Similarly, GB-treated plants under saline conditions showed markedly improved growth parameters due to its capacity to protect photosynthetic machinery and maintain ionic homeostasis [43, 44]. Previous research on ‘Gahrawy’ mango seedlings also found that GB applications, especially when combined with other ameliorants like sodium silicate, significantly improved growth parameters, including leaf number and stem length, by counteracting the negative impacts of salt [4]. Algae Extract (AE) also enhanced growth, particularly under moderate salinity. AE contains bioactive compounds and minerals that enhance nutrient uptake and stimulate vegetative growth under stress [45–47]. This supports earlier findings by *Battacharyya et al.* [48] and *Van Oosten et al.* [48], who reported that seaweed-derived biostimulants contain auxins, cytokinins, betaines, and polyamines that stimulate cell division and elongation under stress conditions. The genotype-specific response observed in this study, where GB was more effective in Gahrawy and AE was more effective in Sokkary under certain salinity levels, suggests differential regulation of osmotic and ionic balance between the two rootstocks, as also noted by [49] in date palm cultivars exposed to salt stress. Stem diameter was less sensitive to salinity than plant height but still showed a significant decline at 3000 ppm. The greater stem diameter promoted by AE in Gahrawy under tap water (10.35 mm) and by GB in Sokkary under severe salinity (8.71 mm) indicates treatment-genotype compatibility in maintaining vascular tissue integrity, potentially through the regulation of cell wall synthesis and secondary growth, as discussed by [50] in the context of biostimulant-mediated growth improvement. Leaf number and leaf area were progressively reduced by increasing salinity, mirroring the findings of [51] in mango seedlings subjected to salinity stress, who attributed reduced leaf area to stomatal closure and impaired cell expansion. The present data showed that AE significantly increased leaf area (77.35 cm²) and preserved it under 1500 ppm (> 78 cm²), likely through its content of alginic acid and growth-promoting phytohormones, consistent with the mechanism described by *Shukla et al.* [52]. Leaf thickness increased under moderate salinity in both rootstocks, a xeromorphic adaptation that reduces transpirational water loss, as previously documented in [53, 54] Sokkary’s consistently thicker leaves suggest a structural adaptation strategy that complements its biochemical stress tolerance responses.

The optical quality index of leaves declined significantly with increasing salinity, with the lowest value recorded in untreated Sokkary at 3000 ppm (2.16). This decline reflects salt-induced degradation of chloroplast structure and pigment breakdown, as described by [12, 55]. Both rootstocks showed declines in physiological parameters under 1500 ppm and 3000 ppm salinity, linked to osmotic stress limiting water availability and the toxic effects of Na⁺ on the photosynthetic apparatus [7, 45, 46]. Both GB and AE improved leaf optical quality under salinity, with AE slightly outperforming GB in Gahrawy at 1500 ppm (4.83 vs. 4.33), which may reflect AE’s dual role in antioxidant support and chlorophyll stabilization. Chlorophyll content (SPAD values) followed a similar pattern, declining under severe salinity while being preserved or enhanced by biostimulant application. GB and induced compatible solutes like proline play a synergistic role in improving salinity tolerance via physiological and biochemical reprogramming [51, 56, 57]. These solutes increase cellular osmotic potential, thereby maintaining relative water content and protecting the integrity of chlorophyll and other photosynthetic enzymes [46, 48]. GB maximized chlorophyll in Gahrawy under tap water (69.60 SPAD), while AE was superior in Gahrawy at 1500 ppm (62.42 SPAD). These results align with the findings by [57, 58], who found that GB protects chloroplast membranes from oxidative damage by scavenging reactive oxygen species (ROS), while seaweed extracts supply micronutrients and amino acids that support de novo chlorophyll biosynthesis [50] reviewed extensively the role of protein hydrolysates and seaweed extracts in improving SPAD values and photosynthetic efficiency under abiotic stress, corroborating the current results. Proline and algal extract treatments in mango have also been shown to promote higher gas exchange and total soluble carbohydrate levels, which support physiological maintenance during stress [45].

The maintenance of a favorable K⁺/Na⁺ ratio is critical for plant survival under salinity, as K⁺ is essential for osmotic regulation, stomatal movement, and enzyme activation [59, 60]. In the present study, Gahrawy rootstock demonstrated a significantly higher leaf K% (2.174%) than Sokkary (1.488%), while Sokkary rootstock accumulated more Na⁺ (1.335% vs. 1.266%), suggesting a superior capacity in Gahrawy for maintaining potassium levels. This ionic profile suggests that Gahrawy possesses more efficient mechanisms for K⁺ retention and Na⁺ exclusion, possibly through enhanced activity of high-affinity K⁺ transporters (HKTs) and vacuolar Na⁺ compartmentalization via the NHX antiporters, as described by [41, 61] Our findings also highlight a significant increase in leaf Na⁺ content under salinity, which often leads to nutrient imbalances and enzymatic inhibition. Foliar GB spray produced the highest mean K% (1.987%), while both GB and AE significantly reduced leaf Na% compared to the unsprayed control. The strongest Na⁺-lowering effect was observed in Sokkary at 3000 ppm, where GB reduced Na% from 2.647 to 1.296%. The ameliorative roles of GB and AE were further evident in their ability to enhance the concentration of N, P, K⁺, Ca²⁺, and Mg²⁺ in leaf tissues [58, 62, 63]. AE, in particular, has been shown to counteract the decline in mineral contents caused by salinity in various crops, likely by improving root system efficiency and nutrient transport [12, 45]. These results are in accordance with *Nawaz & Ashraf* [64] and the more recent findings of *Hameed et al.* [65], who reported that GB application in salt-stressed plants reduced Na⁺ influx by stabilizing plasma membrane integrity and modulating the activity of Na⁺/H⁺ antiporters. GB further supports this balance by protecting the plasma membrane and ion transporters from oxidative damage [4, 66].The role of seaweed extracts in improving K⁺/Na⁺ homeostasis has also been confirmed by [48, 52], that supporting the present findings. Although salinity is generally reported to reduce leaf K⁺ due to Na⁺/K⁺ competition at uptake and transport sites, our data showed the opposite trend, with K% rising from 1.521% to 2.066% alongside increased Na% (0.985–1.656%) as salinity increased. This pattern is consistent with an active K⁺-retention strategy reported in salt-tolerant genotypes, whereby selective transporters such as HKT and SKOR help maintain a favorable K⁺/Na⁺ ratio rather than allowing passive loss of both ions since HKT transporters mediate a conserved mechanism for sodium exclusion and maintenance of a high K+/Na+ ratio in leaves during salinity stress, and because a high K+:Na+ ratio in tissue is an important indicator of greater salt tolerance, achieved through selective ion uptake and transport mechanisms that allow exclusion or compartmentalization of excess Na+ while retaining K+. This supports interpreting the K%/Na% ratio, rather than absolute K% alone, as the more meaningful index of ionic balance in our system, consistent with Gahrawy’s higher K% coinciding with a more favorable K⁺/Na⁺ ratio than Sokkary [67, 68].

Total phenolic content increased significantly at 1500 ppm (3.29 mg g⁻¹ DW) compared to tap water (2.78 mg g⁻¹ DW), consistent with the well-documented induction of secondary metabolic pathways under moderate stress. This hermetic response, where sublethal stress activates defense metabolism, has been described by [6, 62, 69, 70]. The plateau in phenolic content at 3000 ppm, despite increasing salinity, may indicate a saturation of the phenylpropanoid pathway or increased consumption of phenolic compounds for cell wall lignification. This hypothesis warrants further investigation as discussed by [55]. GB application induced the highest phenolic content in Gahrawy at 1500 ppm (3.98 mg g⁻¹ DW) and in Sokkary at 3000 ppm (3.74 mg g⁻¹ DW), indicating rootstock-specific signaling responses. This differential phenolic induction between genotypes under stress has been reported in other tree crops by [4, 10, 49]. Flavonoid content peaked at moderate salinity (19.61 mg g⁻¹ FW), with GB producing the highest values at 1500 ppm in both rootstocks. This finding supports the work of [71, 72], they reviewed that flavonoids, particularly quercetin and kaempferol derivatives, act as secondary antioxidants and blue-light absorbers that protect the photosynthetic apparatus under oxidative stress. The slight superiority of AE in maintaining flavonoids in Gahrawy under severe salinity (18.72 mg g⁻¹ FW) suggests that algae-derived compounds independently support the flavonoid biosynthesis pathway, possibly through the supply of specific amino acid precursors [15, 62].Proline accumulation is a well-documented compatible-solute response in mango under salinity. And the studies revealed indicating a possible role of this compound in mango salinity tolerance closely related with rootstock genotype. Broader screening across mango cultivars has confirmed that significant accumulation of proline, glycine betaine, and total sugars represents a common mechanism employed by tolerant cultivars to counter osmotic stress under saline conditions [8].Proline content showed no significant rootstock or salinity main effects, but unsprayed plants accumulated significantly higher levels (27.2 µmol g^− 1^ FW) than treated plants, indicating that GB and AE alleviated osmotic stress sufficiently to reduce the need for proline-mediated adjustment. This is consistent with the interpretation of proline as a stress distress signal rather than a beneficial osmolyte at high concentrations, as discussed by [73, 74]. they emphasis that proline overaccumulation in untreated plants reflects cellular oxidative damage rather than effective tolerance.

Glycine betaine content in leaves was significantly higher in Gahrawy (2.91 µmol g⁻¹ DW) than in Sokkary (2.20 µmol g⁻¹ DW), and was further enhanced by foliar GB spray under all salinity levels. The synergistic accumulation of endogenous GB following exogenous application is a well-documented phenomenon, attributed to feedback amplification of BADH (betaine aldehyde dehydrogenase) enzyme activity and related metabolic reprogramming [17, 75]. The interaction performance produced by Gahrawy, GB and high salinity combination consistently produced the highest GB leaf content, reinforcing the genotype-dependent capacity for compatible solute accumulation under stress.

The accumulation of reactive oxygen species (ROS), such as H₂O₂, and the resulting lipid peroxidation (measured by MDA levels) are hallmarks of salinity-induced oxidative stress [45, 56]. Malondialdehyde (MDA) levels increased progressively with salinity (from 16.11 to 20.00 µmol g⁻¹ FW), reflecting enhanced lipid peroxidation of membrane phospholipids under oxidative stress. Our results showed significant increases in H₂O₂ and MDA levels in salt-stressed ‘Sokkary’ and ‘Gahrawy’ seedlings, indicating membrane damage. The strongest MDA accumulation was observed under 3000 ppm in untreated plants (up to 24.21 µmol g⁻¹ FW). Both GB and AE significantly reduced MDA accumulation (≈ 16.9 vs. 19.76 µmol g⁻¹ FW in control). These results align with [40, 55], who demonstrated that salinity-induced ROS burst leads to lipid peroxidation cascades that compromise membrane integrity, while compatible solutes and biostimulants counteract this by quenching singlet oxygen and hydroxyl radicals.

Hydrogen peroxide (H₂O₂) accumulation increased with salinity, and interestingly, Gahrawy accumulated significantly more H₂O₂ than Sokkary despite its greater inherent tolerance. This pattern has been explained in the context of controlled ROS signaling, where moderate H₂O₂ accumulation in tolerant genotypes may function as a secondary messenger activating downstream defense pathways, as proposed by *Mitctler et al.* [76]. Both GB and AE effectively reduced H₂O₂ at all salinity levels, with Sokkary showing greater ROS reduction under increasing stress, indicating genotypic differences in oxidative metabolism and enzymatic scavenging efficiency. DPPH radical scavenging activity followed a hermetic pattern, peaking at moderate salinity (46.64%) and declining severely at 3000 ppm (29.64%). Gahrawy exhibited higher baseline DPPH activity (40.23% compared to 37.13% in Sokkary), consistent with its higher phenolic and flavonoid content. AE application produced the highest antioxidant activity in both rootstocks at 1500 ppm (Sokkary: 50.00%; Gahrawy: 53.00%), and meaningfully restored activity under 3000 ppm. The superior performance of AE in DPPH assays may relate to its supply of fucoxanthin, phlorotannins, and sulfated polysaccharides, which are potent hydrogen donors in radical chain termination reactions [77–79].

The rootstocks responded to salinity stress by upregulating antioxidant enzymes, including ascorbate peroxidase (APX), peroxidase (POD), and polyphenol oxidase (PPO). Ascorbate peroxidase (APX) activity was higher in Sokkary (2.66 U(µmol g⁻¹ FW) than Gahrawy (1.99 U (µmol g⁻¹ FW) and peaked at moderate salinity (1500 ppm: 2.60 U(µmol g⁻¹ FW) before declining at 3000 ppm. The observed downregulation of APX under severe stress may reflect enzyme inactivation by excessive H₂O₂ or substrate limitation, as documented by [55]. The decline in APX activity at the highest stress level may be explained, at least in part, by direct oxidative inactivation of the enzyme by excess H_2_O_2_, a mechanism previously reported for APX was clarified by (Miyake and Asada [80] and consistent with the concurrent increase in H_2_O_2_ content observed under this treatment Table 2. Further studies involving direct assessment of enzyme oxidation would help confirm this mechanism. Notably, unsprayed plants showed higher APX activity than treated plants, suggesting that Glycine Betaine and Algae Extracts alleviated oxidative load sufficiently to reduce the enzymatic demand for ROS scavenging, consistent with observations by previous works such as [52, 81–83]. Polyphenol oxidase (PPO) activity showed a contrasting pattern, being higher in Gahrawy (63.14 U(µmol g^− 1^ FW) and increasing progressively with salinity (from 37.49 under tap water to 72.10 at 3000 ppm). The dramatic PPO induction in untreated Gahrawy at 1500 ppm (83.53 U(µmol g^− 1^ FW) suggests a strong oxidative defense activation in this rootstock, which was moderated by anti-saline treatments (GB: 59.50; AE: 60.77 U(µmol g^− 1^ FW). This modulation confirms that biostimulants reduce the cellular oxidative burden requiring PPO intervention, in agreement with [52, 56], who demonstrated that foliar spraying of osmolytes anti-stresses reduces PPO induction in salinity-stressed species by minimizing chloroplast-derived ROS generation. AE has been further reported to significantly increase the activities of SOD, APX, and CAT under salinity, effectively lowering ROS levels and membrane damage [45]. Peroxidase (POD) activity was strongly induced by salinity, reaching 64.53 U µmol g⁻¹ FW at 3000 ppm compared to 24.30 under tap water, and was higher in Gahrawy overall. This salinity-induced POD elevation is a widely reported defense response [55, 76] and reflects the role of POD in oxidizing phenolic substrates and participating in lignification, cell wall strengthening, and H₂O₂ catabolism. Similarly, exogenous biostimulant treatments in mango have been shown to elevate POD and PPO activities, providing a more robust scavenging system for ROS [5]. AE reduced overall POD more than GB (31.02 vs. 41.84 U µmol g⁻¹ FW), though the magnitude of reduction was rootstock- and salinity-dependent. AE was particularly effective in Sokkary at 3000 ppm, reducing POD from 95.1 to 27 U µmol g⁻¹ FW, while GB was more consistently effective in Gahrawy across moderate and high salinity. This upregulation of the antioxidant system is a key mechanism through which AE and GB protect the cell from oxidative damage and maintain membrane stability under high salinity [45, 56]. These contrasting patterns confirm genotype-specific enzymatic regulation of the antioxidant defense network under salinity [10, 49, 56]. Overall, the biochemical responses of the seedlings varied markedly with increasing salinity levels, highlighting the extent of stress-induced metabolic alterations and the differential capacity of the genotypes to cope with salt. The antioxidant capacity of mango (*Mangifera indica* L.) displays a clear biphasic response under increasing salinity. Because mangoes are naturally salt-sensitive, exposure to moderate salinity (1,500 ppm) acts as a mild stress signal that triggers protective defense mechanisms. At this level, the plant upregulates key antioxidant enzymes specifically peroxidase (POD) and ascorbate peroxidase (APX) along with non-enzymatic defenses such as total phenols, flavonoids, and overall DPPH radical-scavenging activity. Exogenous applications of glycine betaine (GB) and seaweed extracts (AE) significantly enhance this defensive capacity. Glycine betaine serves as a protective osmolyte that stabilizes enzyme structures and photosynthetic machinery, while bioactive compounds in seaweed extracts further stimulate the plant’s natural antioxidant synthesis. However, under severe salinity (3,000 ppm), these defensive systems collapse [84, 85]. Extreme salt levels cause severe ion accumulation and water stress, creating excessive reactive oxygen species (ROS) that overwhelm the plant’s scavenging threshold. This extreme stress inactivates key enzymes like APX and POD, depletes essential antioxidant pools, and disrupts broader metabolic pathways needed to produce phenols and flavonoids. Consequently, while biostimulants like GB and seaweed extracts effectively fortify mango defenses under moderate stress, severe salinity leads to critical metabolic failure that overcomes the plant’s antioxidant capacity [12, 84, 86].

### Leaf anatomical adaptations

Salinity induces anatomical modifications in mango leaves to limit water loss and cope with ion toxicity [87]. Leaf anatomical analysis revealed coordinated structural modifications in both mango rootstocks in response to salinity and biostimulant application, with pronounced genotypic differences in plasticity; however, to move beyond a purely descriptive account, each structural shift is interpreted below in relation to its quantitative functional consequence for water relations, gas exchange, and hydraulic transport. In Sokkary, cuticle thickening at 1500 ppm alone (3.1 μm vs. 2.5 μm in control, a 24% increase) represents a typical xeromorphic adaptation that reduces cuticular transpirational water loss under osmotic stress [53, 88]. ]; because cuticular conductance declines approximately in proportion to cuticle thickness, this magnitude of increase is functionally consistent with a meaningful reduction in non-stomatal water loss rather than a marginal structural change. By contrast, GB application at both salinity levels reduced cuticle thickness to 2.1 μm (a 32% reduction relative to the 1500 ppm treatment without GB), suggesting that GB-mediated osmotic adjustment lowers the physiological need for a structural water-retention barrier by maintaining cell turgor biochemically rather than anatomically Acosta-Motos et al. [40]. The shift in the palisade: spongy mesophyll ratio to 53% under 3000 ppm + GB in Sokkary, a 43% increase relative to the 37% control ratio, is functionally significant rather than merely descriptive: leaf-level photosynthetic capacity scales positively with the surface-area-to-volume ratio of the photosynthetically active mesophyll, since Amax increases with increasing spongy mesophyll surface-area-to-volume ratio reflecting a strong positive relationship between the quantity of photosynthetically active mesophyll surface area and leaf-level photosynthetic capacity. This supports the interpretation that GB-induced mesophyll restructuring lowers internal CO₂ diffusive resistance and improves mesophyll conductance, rather than simply yielding a “compact” tissue arrangement [89, 71]. Conversely, 3000 ppm without biostimulants caused spongy tissue hypertrophy (145.1 μm, a 38% increase over control), most plausibly resulting from intercellular space expansion driven by salt-induced cell plasmolysis [7]; functionally, this enlargement increases the diffusion path length for CO₂ without a corresponding gain in absorptive surface area, and can therefore be interpreted as a maladaptive, passive consequence of osmotic injury rather than an active tolerance response — a distinction that becomes interpretable only once the ratio is benchmarked against the GB-treated, functionally optimized architecture described above [90].Vascular bundle dimensions in Sokkary were most enhanced at 3000 ppm alone (453.8 × 510.1 μm), with the thickest xylem (304.9 μm) and the largest xylem vessel diameter (37.7 μm), representing a compensatory strategy to maintain long-distance water transport under reduced turgor [91]. In Gahrawy, vascular bundles were largest under tap water + AE (310.8 × 331.4 μm) and smallest under 3000 ppm alone (191.7 × 203.3 μm), suggesting AE promotes vascular development under non-stress conditions while high salinity without treatment constrains it. The higher K% observed in Gahrawy may be linked to specific anatomical adaptations favoring K⁺ over Na⁺ transport in the root or vascular system. Sclerenchyma development peaked under 1500 ppm + AE in both rootstocks (38.4 μm in Sokkary; 44.5 μm in Gahrawy, representing increases of approximately 22% and 28% over their respective controls), indicating that AE combined with moderate salinity enhances mechanical tissue reinforcement consistent with improved structural resilience and reduced susceptibility to turgor-loss-induced collapse under osmotic stress [92]. Secretory duct dimensions were greatest under 3000 ppm + GB in Sokkary (height 125.1 μm; width 113.2 μm), likely reflecting enhanced production of terpenoids and secondary metabolites as part of the chemical defense response to severe stress [93] ]; this anatomical enlargement is functionally consistent with the concurrent increases in non-enzymatic antioxidant markers (phenolics, flavonoids) discussed above, linking structural secretory capacity to biochemical stress mitigation rather than representing an isolated morphological trait. The midrib region showed differential sensitivity between rootstocks, with maximum midrib width occurring at 3000 ppm alone in both Sokkary (1554 μm) and Gahrawy (1607.9 μm), reflecting compensatory vasculature under diminished hydraulic conductance under severe ionic stress [61]. Pith and cortex enlargement observed predominantly at 3000 ppm without biostimulants in both rootstocks suggests parenchymatous water storage as a passive drought-avoidance mechanism, functioning as a buffering reservoir against transient water deficits rather than an active transport adaptation. GB treatments, by contrast, tended to favor a more compact internal midrib architecture with proportionally larger pith under high salinity, indicating a more active, metabolically driven tolerance strategy in which osmotic adjustment substitutes for passive structural storage. Collectively, these findings indicate that the anatomical modifications observed here are not uniformly adaptive: several (cuticle thickening, sclerenchyma reinforcement, GB-modulated mesophyll compaction) correspond to quantifiable improvements in water conservation, photosynthetic efficiency, or mechanical resilience, whereas others (spongy tissue hypertrophy, vascular constriction in unmitigated Gahrawy) reflect passive consequences of osmotic and ionic injury. This distinction underscores that GB and AE biostimulants play a significant role in preserving leaf structural integrity and enhancing vascular and mesophyll function under salinity stress, thereby contributing to improved growth and physiological performance in both mango rootstocks, and that future work correlating these anatomical traits statistically with gas-exchange and ion-partitioning data would further substantiate the functional linkages proposed here.

Pearson correlation analysis indicated strong associations among salinity-related physiological traits, where Na⁺ accumulation was positively correlated with oxidative stress indicators such as MDA and antioxidant enzymes (PPO and POD), while showing negative relationships with chlorophyll content and plant quality traits, confirming that ionic toxicity is closely linked to oxidative damage and growth suppression under salt stress. Conversely, positive correlations among chlorophyll, leaf traits, and DPPH activity suggest coordinated enhancement of photosynthetic capacity and antioxidant protection, particularly in plants treated with antistress biostimulants such as glycine betaine and algae extract. Principal Component Analysis (PCA) further simplified these relationships by identifying a major stress gradient separating growth-related traits from oxidative stress markers, and a second adaptive axis associated with osmoprotectants and secondary metabolites, reflecting biochemical adjustment mechanisms under salinity. These multivariate patterns are consistent with established statistical interpretations of PCA and correlation analysis [36, 37] and with physiological responses of plants to salinity stress described by Rana Munns and Mark Tester [94, 95]. As detailed in the Materials and Methods, the applied salinity levels reflected nominal irrigation concentrations under a pulsed/intermittent exposure regime rather than a constant root-zone EC. This dynamic pattern more closely simulates field salinity fluctuations, thereby enhancing the ecological realism and field applicability of the observed plant responses. Future studies are recommended to directly monitor root-zone electrical conductivity to further characterize the salinity dynamics experienced by the root system.

## Conclusion

This study confirms that salinity imposes multidimensional stress on mango rootstocks, with tolerance depending on genotype, stress severity, and biostimulant type. Gahrawy showed greater inherent salinity tolerance through improved K⁺ retention, osmotic adjustment, and antioxidant defense. Both rootstocks benefited from biostimulants, although responses varied according to genotype and salinity level. Foliar application of GB was generally more effective in osmotic adjustment, ionic balance, and chlorophyll protection, particularly under severe salinity. AE mainly enhanced antioxidant activity and leaf development under moderate salinity, but neither treatment fully alleviated severe salt stress. Overall, Gahrawy combined with context-specific GB or AE application may provide a practical strategy for sustaining mango production under saline conditions. Future molecular studies on ion transporters and stress-signaling pathways are recommended to clarify the mechanisms underlying these responses.

## Data Availability

The data generated during and/or analysed during the current study are available from the corresponding author on reasonable request.
